# High-Throughput Field Phenotyping Traits of Grain Yield Formation and Nitrogen Use Efficiency: Optimizing the Selection of Vegetation Indices and Growth Stages

**DOI:** 10.3389/fpls.2019.01672

**Published:** 2020-01-17

**Authors:** Lukas Prey, Yuncai Hu, Urs Schmidhalter

**Affiliations:** Chair of Plant Nutrition, Technical University of Munich, Munich, Germany

**Keywords:** phenomics, smart farming, remote sensing, nitrogen use efficiency, yield prediction, red edge, water band indices, breeding

## Abstract

High-throughput, non-invasive phenotyping is promising for evaluating crop nitrogen (N) use efficiency (NUE) and grain yield (GY) formation under field conditions, but its application for genotypes differing in morphology and phenology is still rarely addressed. This study therefore evaluates the spectral estimation of various dry matter (DM) and N traits, related to GY and grain N uptake (Nup) in high-yielding winter wheat breeding lines. From 2015 to 2017, hyperspectral canopy measurements were acquired on 26 measurement dates during vegetative and reproductive growth, and 48 vegetation indices from the visible (VIS), red edge (RE) and near-infrared (NIR) spectrum were tested in linear regression for assessing the influence of measurement stage and index selection. For most traits including GY and grain Nup, measurements at milk ripeness were the most reliable. Coefficients of determination (*R*²) were generally higher for traits related to maturity than for those related to anthesis canopy status. For GY (*R*² = 0.26–0.51 in the three years, *p* < 0.001), and most DM traits, indices related to the water absorption band at 970 nm provided better relationships than the NIR/VIS indices, including the normalized difference vegetation index (NDVI), and the VIS indices. In addition, most indices including RE bands, notably NIR/RE combinations, ranked above the NIR/VIS group. Due to index saturation, the index differentiation was most apparent in the highest-yielding year. For grain Nup and total Nup, the RE/VIS index MSR_705_445 and the simple ratio R780_R740 ranked highest, followed by other RE indices. Among the vegetative organs, *R*² values were mostly highest and lowest for leaf and spike traits, respectively. For each trait, index and partial least squares regression (PLSR) models were validated across years at milk ripeness, confirming the suitability of optimized index selection. PLSR improved the prediction errors of some traits but not consistently the R² values. The results suggest the use of sensor-based phenotyping as a useful support tool for screening of yield potential and NUE and for identifying contributing plant traits—which, due to their expensive and cumbersome destructive determination are otherwise not readily available. Water band and RE indices should be preferred over NIR/VIS indices for DM traits and N-related traits, respectively, and milk ripeness is suggested as the most reliable stage.

## Introduction

Spectral high-throughput sensing has gained increasing attention for efficient assessment of genotypic performance of plant breeding material (Furbank and Tester, 2011; Araus and Cairns, 2014). Various authors have stressed the suitability of using reflectance data measured earlier in the season for the estimation of grain yield (GY). This would enable plant breeders to focus on a limited set of promising genotypes for further rating, thus even making yield determination of dismissed lines unnecessary (Garriga et al., 2017). Besides GY, GY formation, its mechanisms, and the contribution of plant organs were less frequently addressed with spectral methods, especially for the discrimination of genotypes. However, a better understanding of these mechanisms could facilitate plant breeders to target specific traits (Acquaah, 2007), such as leaf area and nitrogen (N) concentration for increasing assimilation, and spike and culm characteristics for increasing the sink and storage size for assimilates, respectively (Schnyder, 1993; Feng et al., 2016).

The contribution of plant organs as sink or source for assimilates and N differs both between genotypes and between growth stages, so that their potential use as traits for indirect selection differs during the grain-filling phase (Barmeier and Schmidhalter, 2017; Prey et al., 2019b; Prey et al., 2019c). Evaluating the variation of these traits in breeding lines can provide valuable complementary information for plant breeders for optimized selection of N use efficiency (NUE), notably GY and grain N uptake (GNup). Additionally, estimating the translocation of dry matter (DM) and N from vegetative organs would contribute to the understanding of promising strategies of the temporal DM and N acquisition (Slimane et al., 2013; Prey et al., 2019a; Prey et al., 2019b) However, the determination of such traits is expensive and cumbersome, thus requiring low-cost robust high-throughput techniques (Nguyen and Kant, 2018).

Such spectral methods need to be optimized in terms of the selection of suitable spectral bands, growth stages for measurements, and spectral models. For GY, different spectral vegetation indices (SVI) were compared for the in-season estimation in durum (Aparicio et al., 2000; Royo et al., 2003), spring barley (Rischbeck et al., 2016; Barmeier et al., 2017), or wheat (Tucker et al., 1980; Raun et al., 2001; Freeman et al., 2003; Moges et al., 2004; Babar et al., 2006a; Babar et al., 2006b; Babar et al., 2007; Prasad et al., 2007a; Prasad et al., 2007b; Gizaw et al., 2016a; Gizaw et al., 2016b). A number of these studies that were often conducted in warm or drought-prone environments strengthened the suitability of band combinations from the near-infrared (NIR) spectrum, including the water absorption band around 970 nm (Babar et al., 2006b; Gutierrez et al., 2010b; Gizaw et al., 2016a; Rischbeck et al., 2016; Becker and Schmidhalter, 2017; Garriga et al., 2017) due to the relation of canopy water mass with biomass and water status with assimilation, respectively, as well as the lower saturation of these bands.

In contrast to GY, GNup and the underlying traits of the formation of GY and GNup were rarely assessed with spectral methods. Barmeier and Schmidhalter (2017) evaluated the spectral estimation of organ-level DM and N uptake (Nup) traits at anthesis and dough ripeness in spring barley and recommended the R780_R670 simple ratio index for DM traits to overcome the saturation of the normalized difference vegetation index (NDVI). The DM and Nup of leaf blades followed by those of culms were mostly better predicted than those of spikes and leaf sheaths. Using red edge (RE)-based SVIs in winter wheat breeding lines grown in small plots, Frels et al. (2018) found mostly weaker but significant relationships with DM and Nup at anthesis and maturity as well as with N harvest index (NHI), N utilization efficiency (NutEff), N uptake efficiency (NupEff), and post-anthesis N uptake (PANup). These authors recommended the RE Maccioni index and identified the early grain filling stage as the most promising. Testing many SVIs for predicting GY, GNup, and NUE traits, Pavuluri et al. (2015) confirmed related indices such as the R780_R740 (Mistele et al., 2004) and found better correlations under reduced N fertilization, that was ascribed to the lower saturation in thinner canopies. Various studies found SVIs to be highly heritable (Babar et al., 2007; Prasad et al., 2007a; Frels et al., 2018) or to be related to QTLs associated with GY (Gizaw et al., 2016a), thus to be used as promising indirect selection tools if sufficient correlations are found early enough in the season. Most studies that assessed traits related to N status in response to N fertilization predominantly agree on the usefulness of RE bands for vegetative N concentration (NC) (Mistele and Schmidhalter, 2008a; Li et al., 2010), Nup (Mistele and Schmidhalter, 2008b; Mistele and Schmidhalter, 2010; Li et al., 2013; Guo et al., 2017; Prey and Schmidhalter, 2019a), N status, biomass, and LAI (Hansen and Schjoerring, 2003), as well as grain NC and Nup (Li et al., 2014; Prey and Schmidhalter, 2019b), due to the shift in the RE reflection as indicator for the N status. RE indices were also found useful for the estimation of biomass traits due to their higher sensitivity in dense canopies (Pavuluri et al., 2015; Frels et al., 2018). Band combinations in the visible range were recommended to be useful for pigment-related traits (Peñuelas et al., 1995; Gitelson et al., 2002; Hansen and Schjoerring, 2003).

Besides SVIs, multivariate analysis such as partial least squares regression (PLSR) holds the advantage of including more spectral information than SVIs, but may be affected by overfitting, so that more calibration data may be required (Mehmood et al., 2012; Overgaard et al., 2013) Comparing SVIs and PLSR, improvements were found for biomass and NC but not for chlorophyll concentration and LAI (Hansen and Schjoerring, 2003). Similar GY predictions were found from PLSR and best SVIs for spring barley (Barmeier et al., 2017). These authors reported improved RMSE values by PLSR but similar coefficients of determination for estimating organ-level traits (Barmeier and Schmidhalter, 2017). While SVIs can be derived from both multi- and hyperspectral data, the potential improvements by multivariate analysis are restricted to hyperspectral data, which comes at the price of more expensive sensors, being less convenient to use in practice, for example on UAV-based platforms (Oehlschläger et al., 2018). Therefore, the benefit of PLSR for traits of NUE and yield formation remains to be evaluated for wheat.

The application of spectral phenotyping depends on the wheat type and environment (Gizaw et al., 2016b). Therefore, the assessment of the influence of measurement conditions regarding growth stage and plant phenology is essential. Yet, often only few growth stages were evaluated for GY, focusing on the period from booting until early grain filling. Several studies reported increasing correlations until milk ripeness (Freeman et al., 2003; Babar et al., 2006a; Gutierrez et al., 2010b; Christopher et al., 2014; Becker and Schmidhalter, 2017). While relationships peaked at anthesis in a rain-fed trial, they increased until maturity under irrigated conditions but were generally lower due to saturation effects in denser canopy (Aparicio et al., 2000). Under water-limited conditions, heading, anthesis (Aparicio et al., 2000; Becker and Schmidhalter, 2017) and stem elongation-heading (Pavuluri et al., 2015) were useful stages. Though year-specific differences were substantial, Frels et al. (2018) recommended the early grain filling stage for NUE traits, but a similar evaluation under high-yielding conditions is missing.

In contrast to the variation driven by altered N application, the detection of variation between genotypes is likely to be more affected by the influence of varying morphology, shifted phenology, and differing contributions of indirect DM and N traits to GY and GNup. Moreover, even if the methods are also promising in high-yielding environments (Gizaw et al., 2016b), many of the studies on spectral GY prediction were conducted on spring wheat (Babar et al. 2006a; Babar et al., 2007; Gutierrez et al. 2010a; Sultana et al., 2014) or in environments with low yield potential. This limits the transferability to high-yielding winter wheat, given that weaker relationships were often reported from irrigated trials or denser canopies compared to drought-stress trials (Aparicio et al., 2000; Babar et al., 2006a; Becker and Schmidhalter, 2017; Frels et al., 2018). Moreover, the optimized selection of SVIs depends on the availability of suitable sensors—characterized by band number, narrowness, and placement—and measurement stages (Thenkabail et al., 2000; Prey and Schmidhalter, 2019b).

The present study, therefore, tested the performance of 48 SVIs for the estimation of GY, GNup, and 45 further organ- and plant-level DM and N traits from hyperspectral passive proximal canopy sensing acquired from leaf development until dough ripeness over three years in a high-yielding West-European environment, based on a previous evaluation of the included DM and N reference traits (Prey et al., 2019b). The questions addressed are (i) the detectability of reference traits, (iii) the influence of optimized selection of specific SVIs, (iii) the selection of optimum growth stages for measurements, and (iv) the use of PLSR in comparison to SVIs.

## Materials and Methods

### Field Experiments and Plant Sampling

The field experiment was conducted over three years from 2014/2015 to 2016/2017 for evaluating traits with influence on NUE and yield formation in a diverse population of winter wheat double haploid breeding lines. The population's parents consisted of elite cultivars and breeding lines provided by regional plant breeders. The population had undergone pre-selection, which removed genotypes peculiar in terms of extreme flowering date, plant height, and disease susceptibility. The trials comprised 75 lines in two replicates in 2014/2015, 75 lines in four replicates in 2015/2016, and 32 selected lines representing the overall yield variation in 4 replicates in 2016/2017. In addition, three high performance cultivars were included as references. The plot width was 1.5 m, and the plot length was 6.5 m. The trial was located approximately 25 km North of Munich (48.406 N, 11.692 E). The soil consisted mainly of homogeneous Cambisols of loamy clay. The precipitation in the main wheat growing period from October to August was 714 mm in 2014/15, 746 mm in 2015/16 and 690 nm in 2016/2017. During the grain filling period in 2015, heat and lack of precipitation caused moderate drought stress whereas grain filling in 2016 was influenced by fungal pathogens. The preceding crop was winter wheat in the first and second year and grass-clover in the third year.

Biomass sampling was conducted at anthesis (Zadoks growth stage 6), and at physiological maturity (stage 9). Sampling dates were determined for each genotype by visual scoring. For sampling at anthesis, 20 randomly selected spiked culms were cut directly at stem base in 2015 and 30 culms in 2016 and 2017, and at maturity 30 culms in 2015 and 50 culms in 2016 and 2017, respectively. The plants were manually separated into leaf blades, stems including leaf sheaths, and spikes. In 2016, only a subset of 34 genotypes was separated into vegetative organs. At maturity, spikes were threshed. Plant samples were oven-dried at 50°C until constant weight was reached and DM weight was determined by weighing. N concentration (NC) of the plant material was analyzed by means of NIR spectroscopy in a Foss Rapid Content Analyzer for leaves and spikes and in a Bruker Vector 22/N for the remaining organs. Final GY per plot was determined using a combined harvester. Spike density per plot was calculated by dividing GY per area by yield per spike. Nitrogen uptake (Nup) was calculated by multiplying DM with NC. Further indirect traits related to yield components, DM and N translocation and N uptake and utilization efficiency were calculated (**Table 1**). Reference traits were categorized into DM and N traits (Prey et al., 2019b). Moreover, these are either direct traits, which were directly retrieved from plant sampling either at anthesis or maturity like DM per ha, NC or Nup, or indirectly derived DM and N traits, which were predominantly calculated using data from both sampling dates or from different plant organs (**Table 1**). See Prey et al. (2019b) for details on the plant sampling, descriptive statistics and correlations of the plant traits. Plant height had been included in the analysis of the reference traits, but was not considered in the spectral analysis since it is easily assessable from height sensors (Barmeier et al., 2016), which were not available on all measurement dates.

**Table 1:** List of traits considered for testing relationships with spectral indices.

Trait group	Trait name	Abbreviation
Dry matter (DM) [kg DM ha^–1^]	Total DM at anthesis	DM Ant
	Leaves DM at anthesis	DM leaves Ant
	Culms DM at anthesis	DM culms Ant
	Spikes DM at anthesis	DM spikes Ant
	Total DM at maturity	DM Mat
	Leaves DM at maturity	DM leaves Mat
	Culms DM at maturity	DM culms Mat
	Chaff DM at maturity	DM chaff Mat
	Grain DM at maturity (grain yield [GY])	DM grain Mat
N concentration (NC) [%]	Leaves NC at anthesis	NC leaves Ant
	Culms NC at anthesis	NC culms Ant
	Spikes NC at anthesis	NC spikes Ant
	Leaves NC at maturity	NC leaves Mat
	Culms NC at maturity	NC culms Mat
	Chaff NC at maturity	NC chaff Mat
	Grain NC at maturity (GNC)	NC grain Mat
Nitrogen uptake (Nup) (kg N ha^–1^)	Total Nup at anthesis	Nup Ant
	Leaves Nup at anthesis	Nup leaves Ant
	Culms Nup at anthesis	Nup culms Ant
	Spikes Nup at anthesis	Nup spikes Ant
	Total Nup at maturity	Nup Mat
	Leaves Nup at maturity	Nup leaves Mat
	Culms Nup at maturity	Nup culms Mat
	Chaff Nup at maturity	Nup chaff Mat
	Grain Nup at maturity (GNup)	Nup grain Mat
	Straw Nup at maturity	Nup straw Mat
derived DM traits	Spike density	spike density
	Grain number per spike	GNS
	Thousand kernel weight	TKW
	DM translocation efficiency	DMTEff
	DM translocation	DMT
	Post-anthesis assimilation	PAA
	Contribution of post-anthesis assimilation to grain filling	CPAA
	Total N utilization efficiency	NutEff_total
	Harvest index	HI
	Grain N utilization efficiency	NutEff_grain
derived N traits	Contribution of post-anthesis N uptake to total N uptake	CPNup
	N harvest index	NHI
	Total N translocation	NT
	Leaves N translocation	NT leaves
	Culms N translocation	NT culms
	Spikes N translocation	NT spikes
	N translocation efficiency	NTEff
	Post anthesis N uptake	PANup
other	Flowering days in June	flowering

### Spectral Measurements

Spectral measurements were conducted using the *PhenoTrac 4* multi-sensor platform during various growth stages throughout the season (**Table 2**). In 2015, measurements were performed on only four dates, in 2016 on 12 dates and in 2017 on 10 dates, with the highest frequency during the grain filling phase due to the more rapid canopy development and the expected better relationships with maturity traits. The *PhenoTrac 4* is equipped with a hyperspectral bidirectional passive point sensor spectrometer (tec5, Oberursel, Germany), measuring at a nominal resolution of approximately 3.3 nm between 300 and 1000 nm. The measurement distance was approx. 80 cm above the canopy. Measurements were registered at a frequency of 5 Hz together with the GPS coordinates from the TRIMBLE RTK-GPS (real-time kinematic global positioning system; Trimble, Sunnyvale, CA, USA). See Kipp et al. (2014) and Erdle et al. (2011) for further description of the sensor system.

**Table 2:** Heritability of indices averaged by index groups by measurement dates calculated for all measurement dates.

Date	d.a.s.	GDD	Growth stage	Heritability (H²)						
				NIR	NIR_VIS	VIS	NIR_RE	NIR_RE_VIS	RE	RE_VIS
2015										
150424	171	194	Tillering	0.29	0.29	0.53	0.32	0.16	0.27	0.29
150625	233	782	Late milk	0.39	0.69	0.86	0.61	0.70	0.61	0.73
150707	245	978	Soft dough	0.52	0.66	0.85	0.70	0.75	0.72	0.72
150716	254	1101	Hard dough	0.74	0.78	0.92	0.76	0.76	0.76	0.77
2016										
160405	175	200	Leaf development	0.59	0.59	0.64	0.56	0.60	0.61	0.59
160411	181	217	Tillering	0.58	0.64	0.70	0.53	0.52	0.56	0.60
160421	191	254	Tillering	0.77	0.78	0.89	0.59	0.62	0.66	0.75
160518	218	385	Stem elongation	0.81	0.50	0.72	0.64	0.68	0.60	0.64
160529	229	487	Booting	0.91	0.66	0.77	0.86	0.86	0.80	0.80
160610	241	615	Anthesis	0.91	0.86	0.91	0.89	0.90	0.86	0.88
160614	245	655	Early milk	0.91	0.88	0.93	0.84	0.91	0.87	0.91
160623	254	766	Milk	0.85	0.83	0.94	0.89	0.90	0.86	0.89
160628	259	839	Late milk	0.84	0.85	0.94	0.91	0.92	0.90	0.89
160708	269	969.5	Early dough	0.78	0.85	0.93	0.89	0.91	0.87	0.87
160710	271	1001	Soft dough	0.83	0.80	0.94	0.88	0.90	0.83	0.83
160719	280	1116	Hard dough	0.74	0.90	0.92	0.90	0.90	0.88	0.87
2017										
170331	159	109	Leaf development	0.77	0.77	0.71	0.74	0.75	0.77	0.77
170413	172	167	Tillering	0.70	0.77	0.81	0.77	0.72	0.77	0.77
170517	206	297	Stem elongation	0.85	0.59	0.66	0.82	0.85	0.81	0.69
170525	214	372	Booting	0.82	0.15	0.43	0.83	0.80	0.72	0.45
170608	228	534	Anthesis	0.94	0.91	0.88	0.93	0.94	0.92	0.87
170614	234	609	Anthesis	0.92	0.40	0.65	0.91	0.87	0.80	0.66
170621	241	706	Milk	0.82	0.33	0.65	0.90	0.86	0.79	0.66
170701	251	862	Early dough	0.86	0.72	0.87	0.90	0.93	0.90	0.83
170705	255	915	Soft dough	0.58	0.89	0.94	0.93	0.95	0.94	0.92
170711	261	1017	Hard dough	0.96	0.96	0.96	0.95	0.95	0.95	0.95

SVIs are grouped according to the included spectral regions (**Table 3**; **Supplementary Figure 1**). See **Supplementary Table 2** for heritability values by indices. Dates of are listed as year/month/day with days after sowing (d.a.s.) and growing degree days based on a 5°C threshold.

### Selection of Vegetation Indices

SVIs were selected from the literature based on previous work that identified useful applications of the indices, and from an Index-database (https://www.indexdatabase.de; Henrich et al., 2012). The indices were grouped by the included spectral ranges (visible light [VIS], the extended RE, and NIR), with the VIS < 700 nm, RE: 700–765 nm and the NIR > 765 nm (**Table 3**; **Supplementary Figure 1**). Prior to index calculation, the spectra were smoothed using a five-band moving average filter (Mistele and Schmidhalter, 2010) in order to remove spectral noise. However, comparisons with unsmoothed data suggested little influence of spectral noise on the trait/index relationships.

**Table 3:** List of spectral vegetation indices considered in this study.

Index	Full name	Spectral band group	Equation	Reference (IDB: Index database)
NWI-1	Normalized water index 1	NIR	(*R*970 − *R*900)/(*R*970 + *R*900)	(Babar et al., 2006b; Babar et al., 2007)
NWI-2	Normalized water index 2	NIR	(*R*970 − *R*850)/(*R*970 + *R*850)	(Babar et al., 2006b; Babar et al., 2007)
NWI-3	Normalized water index 3	NIR	(*R*970 − *R*920)/(*R*970 + *R*9200)	(Babar et al., 2007)
NWI-4	Normalized water index 4	NIR	(*R*970 − *R*880)/(*R*970 + *R*880)	(Babar et al., 2007)
NWI-5	Normalized water index 5	NIR	(*R*970 − *R*930)/(*R*970 + *R*930)	(Prey and Schmidhalter, 2019b)
WBI	Water band index	NIR	*R*900/*R*970	(Peñuelas et al., 1993)
EVI	Enhanced vegetation index	NIR, VIS	$2.5*\frac{\left(R864-R670\right)}{\left(R864+6*R670-7.5*R420+1\right)}$	(Huete et al., 2002)
GNDVI	Green NDVI	NIR, VIS	(*R*780 − *R*550)/(*R*780 + *R*550)	(Gitelson et al., 1996a)
MCARI1	Modiﬁed chlorophyll absorption in reﬂectance index 1	NIR, VIS	1.2∗(2.5(*R*800 − *R*670) − 1.3∗(*R*800 − *R*550))	(Haboudane et al., 2004)
MCARI2	Modiﬁed chlorophyll absorption in reﬂectance index 2	NIR, VIS	$1.5*\frac{\left(2.5*\left(R800-R670\right)-1.3*\left(R800-R550\right)\right)}{(\left({\left(2*R800+1\right)}^{2}-\left(6*R800-5*R670^{0.5}\right)-0.5^{0.5}\right)}$	(Haboudane et al., 2004)
MSAVI	Modified soil-adjusted vegetation index	NIR, VIS	(2∗*R*800 + 1 − (((2∗*R*800 + 1)^2^ − 8∗(*R*800 − *R*670))^0.5^))/2	(Broge and Leblanc, 2001)
MSR(MSR670)	Modified simple ratio 670	NIR, VIS	$\frac{\left(R800/R670-1\right)}{\left({\left(R800/R670+1\right)}^{0.5}\right)}$	(Chen, 1996)
MTVI2	Modified triangular vegetation Index 2	NIR, VIS	$\frac{1.5(1.2*(R800-R550)-2.5*(R670-R550))}{\left({\left({\left(2*R800+1\right)}^{2}-(6*R800-5*R670^{0.5}-0.5)\right)}^{0.5}\right)}$	(Haboudane et al., 2004)
NDVI1	Normalized difference 1	NIR, VIS	(*R*864 − *R*670)/(*R*864 + *R*670)	(Cristiano et al., 2010)
NDVI2	Normalized difference 2	NIR, VIS	(*R*780 − *R*670)/(*R*780 + *R*670)	(Erdle et al., 2013a; Erdle et al., 2013b)
NDVI3	Normalized difference 3	NIR, VIS	(*R*900 − *R*670)/(*R*900 + *R*670)	(Zhao et al., 2004)
OSAVI	Optimized soil-adjusted vegetation index	NIR, VIS	$\frac{\left(1+0.16)*(R800-R670\right)}{\left(R800+R670+0.16\right)}$	(Li et al., 2010)
PSSR	Pigment specific simple ratio	NIR, VIS	*R*800/*R*500	(Ustin et al., 2009)
R780/R550		NIR, VIS	*R*780/*R*550	(Takebe et al., 1990)
R780/R670		NIR, VIS	*R*780/*R*670	(Pearson and Miller, 1972)
WDRVI	Wide dynamic range vegetation index	NIR, VIS	$\frac{\left(0.1*R780-R670\right)}{\left(0.1*R780+R670\right)}$	(Gitelson, 2004)
ARI	Anthocyanin reflectance index	VIS	1/*R*550 − 1/*R*700	(Gitelson et al., 2001)
BGI	Blue green pigment index	VIS	*R*450/*R*550	(Zarco-Tejada et al., 2005)
BRI	Blue red pigment index	VIS	*R*450/*R*690	(Zarco-Tejada et al., 2005)
PRI	Photochemical reflectance index	VIS	(*R*531 − *R*570)/(*R*531 + *R*570)	(Peñuelas et al., 1995)
VARIgreen	Visible atmospherically resistant vegetation index green	VIS	(*R*550 − *R*670)/(*R*550 + *R*670 − *R*470)	(Gitelson et al., 2002)
NDRE	Normalized difference NIR/Red edge index	NIR, RE	(*R*790 − *R*720)/*R*790 + *R*720)	(Barnes et al., 2000)
R780/R740		NIR, RE	*R*780/*R*740	(Mistele et al., 2004)
NDRE_770_750		NIR, RE	(*R*770 − *R*750)/(*R*770 + *R*750)	(Prey and Schmidhalter, 2019b)
R787/R765		NIR, RE	*R*787/*R*765	(Fava et al., 2009)
LCI	Leaf chlorophyll index	NIR, RE, VIS	(*R*850 − *R*710)/(*R*850 + *R*680)	IDB
Maccioni	Maccioni index	NIR, RE, VIS	(*R*780 − *R*710)/(*R*780 − *R*680)	(Maccioni et al., 2001)
REIP	Red edge inflection point	NIR, RE, VIS	$700+40*\frac{\left(\frac{R670+R780}{2}\right)-R700}{\left(R740-R700\right)}$	(Guyot et al., 1988)
TCARI/OSAVI		NIR, RE, VIS	$3*\frac{\left(\left(R700-R670\right)-0.2*\left(R700-R550\right)*R700/R670\right)}{\left(\left(1+0.16\right)*\left(R800-R670\right)/\left(R800+R670+0.16\right)\right)}$	(Haboudane et al., 2002)
HVI	Hyperspectral vegetation index	RE	*R*750/*R*700	(Gitelson et al., 1996b)
R760/R730		RE	*R*760/*R*730	(Mistele and Schmidhalter, 2010; Jasper et al., 2009)
RVSI	Red edge vegetation stress index	RE	(*R*714 + *R*752)/2 − *R*733	(Merton, 1998)
VOG1	Vogelmann 1	RE	*R*740/*R*720	(Vogelmann et al., 1993)
VOG2	Vogelmann 2	RE	(*R*734 − R747)/(*R*715 + *R*726)	(Vogelmann et al., 1993)
DD	Double difference index	RE, VIS	(*R*749 − *R*720) − (*R*701 − *R*672)	(Le Maire et al., 2004)
MCARI	Modiﬁed chlorophyll absorption in reﬂectance index	RE, VIS	((*R*700 − *R*670) − 0.2∗(*R*700 − *R*550))∗(*R*700/*R*670)	(Daughtry et al., 2000)
MND_750_705	Modified normalized difference 750/705	RE, VIS	$\frac{\left(R750-R705\right)}{\left(R750+R705-2*R445\right)}$	(Sims and Gamon, 2002)
MSR_705_445	Modified simple ratio 705/445	RE, VIS	(*R*750 − *R*445)/(*R*705 − *R*445)	(Sims and Gamon, 2002)
MTCI	MERIS terrestrial chlorophyll index	RE, VIS	(*R*750 − *R*710)/(*R*710 − *R*680)	(Dash and Curran, 2003)
NDVI4	Normalized difference 4	RE, VIS	(*R*750 − *R*705)/(*R*750 + *R*705)	(Gitelson and Merzlyak, 1994)
PSRI	Plant senescence reflectance index	RE, VIS	(*R*680 − *R*500)/*R*750	(Sims and Gamon, 2002)
R730/R670		RE, VIS	*R*730/*R*670	(Mistele and Schmidhalter, 2010)
R760/R670		RE, VIS	*R*760/*R*670	(Erdle et al., 2011)

No full names are available for simple ratio indices named by their spectral bands. “R” denotes the reflection in indicated wavebands.

### Statistical Analysis

For each sampling stage, each SVI was tested in simple linear regressions with DM and N-traits using mean values per genotype as averaged across the replicates. Data analysis was conducted in *R* (version 3.4.; R Core Team, 2017), using the *lm*-function. The coefficient of determination (R²) was used to compare the linear relationships. Broad-sense heritability (H²) was calculated for the SVIs for each measurement date using the *lmer* function as H² = Vg/(Vg+Ve/nR), where V denotes the variance component for the effects of genotype (Vg) and of the residual variance (Ve), and nR the number of replicates (nR = 2 in 2015 and nR = 4 in 2016 and 2017).

In order to overcome the influence of differing growing conditions as well as of the date-specific index rankings, indices were quantitatively ranked by their normalized performance for each trait, adapting the ranking by Frels et al. (2018). Since the coefficient of determination is range-dependent but independent of the level of the trait, it represents a bivariate ranking of the genotypes. Therefore, the R² values were used instead of the RMSE values. For each trait, the seasonally mean (**Supplementary Equation 1a**) and maximum (**Supplementary Equation 1c**) R² values of each index were normalized (**Supplementary Equation 1e, f**) to the trait-specific seasonally mean (**Supplementary Equation 1b**) and maximum (**Supplementary Equation 1d**) R² within each year as calculated from the results of all indices, respectively. Thus, a value > 1 indicated a comparative advantage of the index for the trait under consideration. Consequently, both the within-year mean- and maximum-based rankings (**Supplementary Figure 9**) were summed up across the three years for achieving year-independent rankings (**Supplementary Equation 1g, h**). These across-years mean- and maximum- based rank sums were combined by summing up both ranking sums for a unique ranking per trait (**Supplementary Equation 1i**). Considering a selection of indices that is robust towards date-specific effects as more important, the mean-based rank sums were double-weighted. These weighted mean/maximum-rank sums (WMMRS) were used for identifying one trait-specific optimum index, irrespective of the R² level achieved.

The selected indices were validated in test set validations across years by linear regression on the trait-specific WMMRS-indices and on the NDVI (“NDVI2”) and REIP indices, considered as widely used “reference” indices, in comparison to PLSR models. Based on the seasonal evaluation of the SVI-relationships, calibration and validation was conducted using milk ripeness measurements (June 25, 2015, June 28, 2016, and June 21, 2017). Initial PLSR models were fitted on smoothed spectral data for evaluating influential spectral bands. PLSR models used for predictions were based on spectra additionally pretreated by Savitzki-Golay first order derivation due to significant improvements (not shown). Bands below 370 nm and above 990 nm were not included due to spectral noise. PLSR was fitted using the kernel algorithm (Mevik and Wehrens, 2007) in the *pls* package. The optimum number of components was determined by minimizing the cross validation RMSE with the restriction that an additional component further decreased the RMSE by at least 1%. For both SVIs and PLSR, validation was conducted cross-wise on the data of both other years, resulting into each six validation cases.

## Results

### Heritability of Vegetation Indices

Heritability (H²) estimated for all SVIs was higher in 2016 and 2017 than in 2015 and generally increased in all index groups with ongoing plant development (**Table 3**; **Supplementary Table 1**). For all measurement dates in 2015, the group of VIS indices reached the highest H² values, whereas the NIR/RE/VIS indices yielded similar values in 2016, mostly followed by the group of RE/VIS indices. In 2016, unlike in most other groups, the H² of NIR indices was highest (0.90) already at booting and anthesis, followed by decreasing values until hard dough (0.76). Though moderate H² values (~0.60) were already reached before stem elongation, H² mostly exceeded 0.80 only after booting/anthesis, both in 2016 and 2017. Notably, many indices including VIS bands were not heritable during booting, later anthesis and milk ripeness in 2017, whereas most indices with NIR bands still reached high values (>0.80) on these days.

### The Seasonal Trait Assessment

The relationships found between reference traits and SVIs differed between measurement days and between years, so that an identification of optimal measurement dates and SVIs is necessary. Mean and maximum (**Supplementary Figure 2**) coefficients of determination (R²) peaked during milk ripeness and early dough ripeness at the end of June for most traits in both 2015 and 2016, whereas R² values increased for later measurement dates in 2017 (**Figure 3**; **Figure 4**). In all years and for most traits, the steepest R² increase was found between anthesis and milk ripeness, whereas useful relationships were rarely found during the vegetative phase.

For direct DM and Nup traits and for NC, predominantly closer relationships were observed for the maturity traits than for the anthesis traits. Due to the dominant effect of the measurement date, no clear differences in the date suitability by trait were found (**Supplementary Figure 2**). In all years, the VIS indices represented the weakest index group and their *R*² values decreased earlier during grain filling than those of most other indices (**Supplementary Figure 3**). In 2016, several indices with RE bands yielded higher R² values during stem elongation (May 18) than those of the other groups. In 2017, the relationships reached from NIR/VIS indices increased later at anthesis/grain filling than from the other indices.

### Seasonal Relationships and Index Rankings

**Figure 1:**
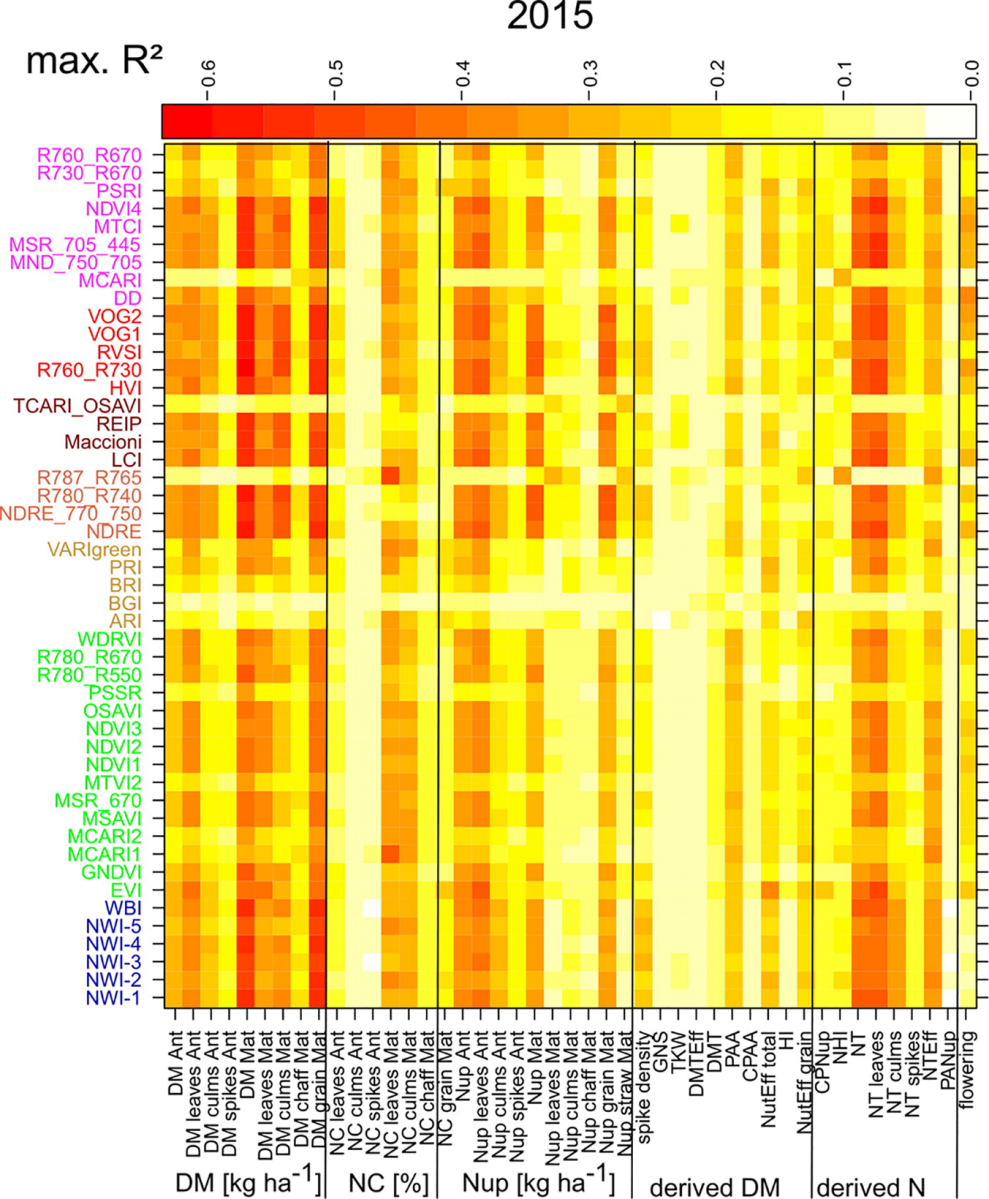
Maximum coefficients of determination (R²) calculated across the R² values of the different measurement dates in 2015 by trait/SVI combinations. Indices are colored according to the included spectral regions (**Supplementary Figure 1**; from bottom to top): NIR (blue), NIR/VIS (green), VIS (orange), NIR/RE (light red), NIR/RE/VIS (brown), RE (red), RE/VIS (purple). Refer to **Supplementary Figure 4** for results of the other years.

**Figure 2:**
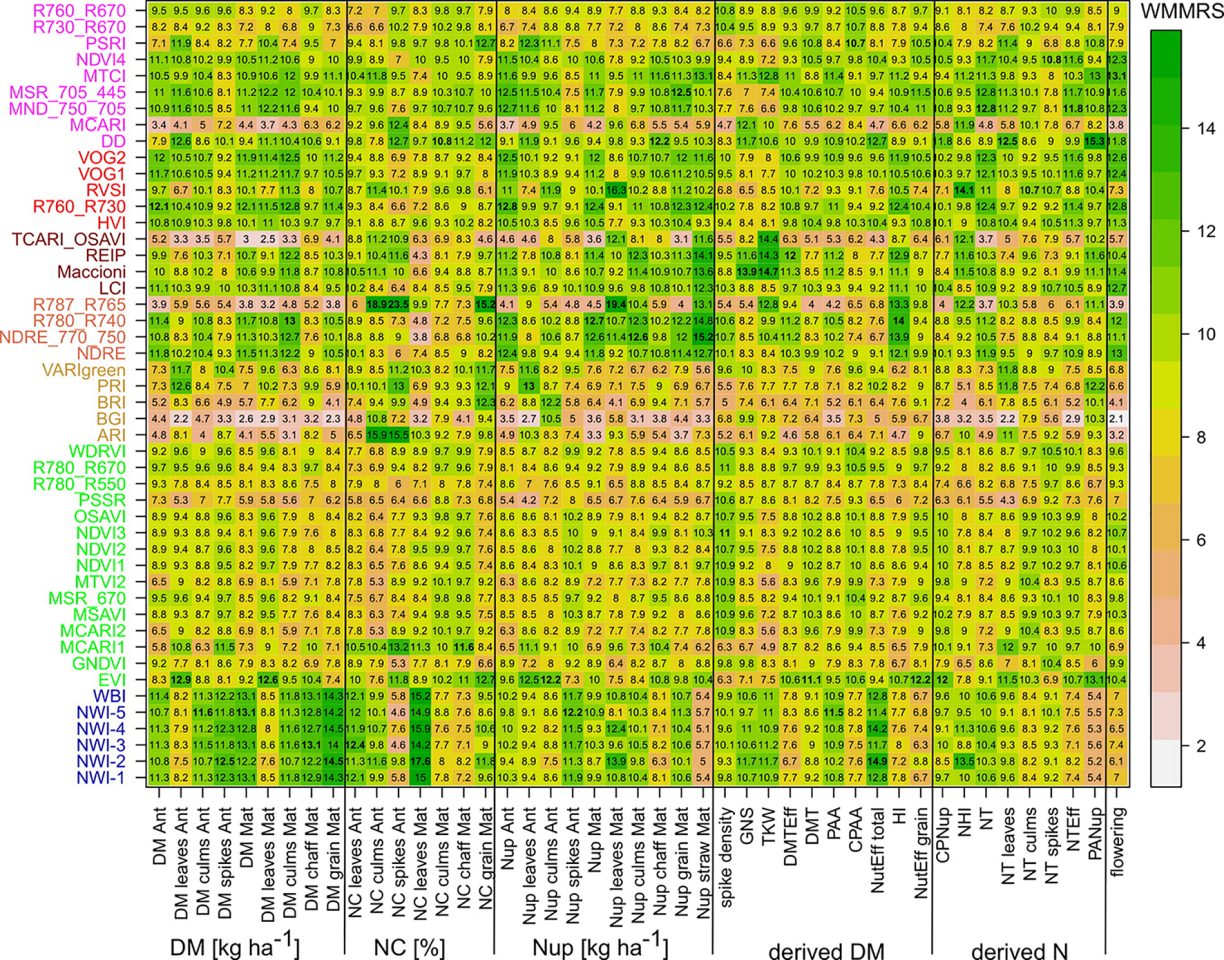
Weighted mean/maximum rank sums (WMMRS) for all SVI/trait combinations. The column-wise and overall WMMRS-mean is 9. A WMMRS > 9 indicates a comparative advantage of an index for a specific trait. WMMRS values are colored from low (white) to high (green) values. For each trait, the highest value is highlighted in bold. For a better comparison in the main range, the color shading for WMMRS > 15 is not differentiated. Indices are colored according to the included spectral regions (Refer to caption of **Figure 1** and **Supplementary Figure 1**).

For assessing the trait detection, the trait-specific index suitability, and the stability over time, seasonally maximum (**Figure 1**; **Supplementary Figure 4**) and mean (**Supplementary Figure 5**) R² values were calculated for each SVI–trait combination across measurement dates in the individual years. The group of direct DM traits was relatively best assessed, followed by direct Nup traits, whereas the derived DM traits were the least estimated (**Table 4**). The relationships differed more strongly between traits and years for the groups of derived DM and N traits. Mean and maximum *R*² values by traits were closely related for most traits, indicating that the comparison of the trait estimation was not derived from specific dates only. For each trait, the indices were ranked based on weighted mean/maximum-rank sums (WMMRS) achieved over the three years (**Figure 2**). Seasonal R² values are presented for selected DM and N traits.

**Table 4:** Best trait-specific indices identified based on the weighted mean/maximum rank sums (WMMRS) and their coefficients of determination (R²; *: *p* < 0.05; **: *p* < 0.01; ***: *p* < 0.001) on the optimum dates.

				R² (within years; rank-based SVI)						Best date						R² (validation)				RMSE (Validation)			
Trait group	Trait	Rank-based best SVI	Ranking (WMMRS)	2015		2016		2017		2015	2016	2017				PLSR	WMMRS-SVI	NDVI2	REIP	PLSR	WMMRS-SVI	NDVI2	REIP
DM (kg DM ha^–1^)	Total DM at anthesis	R760_R730	12	0.33	***	0.11	**	0.33	***	06/25	05/18	07/11	778	814	642	0.18	0.18		0.19	2968	3726		3676
	Leaves DM at anthesis	EVI	13	0.39	***	0.29	**	0.44	***	06/25	06/23	07/11	153	194	178		0.25	0.16	0.18		849	855	825
	Culms DM at anthesis	NWI-5	12	0.18	***	0.08		0.34	***	06/25	06/10	07/01					0.14		0.20		1774		2200
	Spikes DM at anthesis	NWI-2	12	0.11	**	0.23	**	0.18	*	06/25	06/14	06/21	197	163	197		0.12				647		
	Total DM at maturity	NWI-5	13	0.41	***	0.37	***	0.34	***	06/25	07/10	07/05	880	739	840	0.43	0.36	0.23	0.35	3262	3090	4615	4380
	Leaves DM at maturity	EVI	13	0.37	***	0.46	***	0.52	***	06/25	06/28	07/11	95	103	128	0.25	0.36	0.22	0.26	429	412	451	411
	Culms DM at maturity	R780_R740	13	0.45	***	0.42	***	0.29	***	06/25	06/23	05/17	310	381	420	0.37	0.35	0.18	0.36	1689	1934	1829	1941
	Chaff DM at maturity	NWI-3	13	0.18	***	0.21	**	0.10		07/16	07/08	06/21	213	145	178		0.12				522		
	Grain DM at maturity (GY)	NWI-2	15	0.51	***	0.26	***	0.27	**	06/25	06/28	06/21				0.33	0.35	0.20	0.26	1609	1891	2248	2124
N concentration (NC) [%]	Leaves NC at anthesis	NWI-3	12	0.09	**	0.10		0.17	*	06/25	04/05	06/08											
	Culms NC at anthesis	R787_R765	19	0.07	*	0.09		0.23	**	04/24	06/28	06/08											
	Spikes NC at anthesis	R787_R765	23	0.11	**	0.08		0.10		04/24	04/21	07/05											
	Leaves NC at maturity	NWI-2	17	0.32	***	0.42	***	0.21	**	07/07	06/14	07/05				0.13	0.24	0.13	0.09	0.48	0.23	0.26	0.25
	Culms NC at maturity	DD	11	0.26	***	0.18	*	0.22	**	07/07	07/19	07/05					0.09				0.10		
	Chaff NC at maturity	MCARI1	12	0.10	**	0.26	**	0.27	**	07/07	05/18	07/11											
	Grain NC at maturity (GNC)	R787_R765	15	0.07	*	0.08	*	0.08		07/07	07/10	03/31											
Nup (kg N ha^–1^)	Total Nup at anthesis	R760_R730	13	0.37	***	0.14	***	0.33	***	06/25	05/18	07/11				0.14	0.19		0.20	66	77		77
	Leaves Nup at anthesis	PRI	13	0.32	***	0.31	***	0.30	***	06/25	06/23	07/01					0.21	0.16	0.21		30	37	35
	Culms Nup at anthesis	EVI	12	0.19	***	0.09		0.16	*	06/25	06/10	07/11							0.14				31
	Spikes Nup at anthesis	NWI-5	12	0.13	**	0.20	**	0.10		06/25	06/14	05/25											
	Total Nup at maturity	R780_R740	13	0.43	***	0.19	***	0.36	***	06/25	07/10	07/05				0.37	0.32	0.16	0.31	27	66	71	66
	Leaves Nup at maturity	R787_R765	19	0.28	***	0.19	*	0.14	*	07/07	04/21	05/25					0.10				5		
	Culms Nup at maturity	NDRE_770_750	13	0.13	***	0.23	**	0.38	***	04/24	07/08	07/05				0.17	0.17		0.17	6	9		9
	Chaff Nup at maturity	DD	12	0.06	*	0.19	**	0.26	**	07/16	07/10	07/11											
	Grain Nup at maturity (GNup)	MSR_705_445	13	0.31	***	0.21	***	0.37	***	06/25	06/28	06/14				0.27	0.29	0.16	0.30	33	51	56	52
	Straw Nup at maturity	NDRE_770_750	15	0.17	***	0.23	***	0.37	***	07/16	07/10	07/05				0.17	0.11		0.11	12	17		17
derived DM traits	Spike density	NDVI3	11	0.16	***	0.05	*	0.14	*	06/25	06/23	07/05											
	Grain number per spike	Maccioni	14	0.04		0.06	*	0.06		07/16	06/14	04/13											
	Thousand kernel weight [g]	Maccioni	15	0.15	***	0.07	*	0.12	*	07/16	04/05	07/11											
	DM translocation efficiency	REIP	12	0.03		0.19	***	0.17	*	07/07	07/08	03/31											
	DM translocation [kg ha^–1^]	EVI	11	0.08	*	0.20	***	0.07		04/24	07/08	04/13					0.06				1043		
	Post-anthesis assimilation [kg ha^–1^]	NWI-5	12	0.23	***	0.34	***	0.12	*	07/07	07/10	03/31				0.19	0.17	0.14	0.15	1187	1589	1963	1820
	Contribution of post-anthesis assimilation to grain filling	PSRI	11	0.05	*	0.28	***	0.09		04/24	07/08	06/08											
	Total N utilization efficiency	NWI-2	15	0.25	***	0.12	**	0.16	*	06/25	07/19	05/17					0.13				12.69		
	Harvest index	R780_R740	14	0.09	**	0.10	**	0.30	***	07/16	07/10	07/11				0.15				0.06			
	Grain N utilization efficiency	EVI	12	0.21	***	0.14	***	0.21	**	06/25	07/19	07/05											
derived N traits	Contribution of post-anthesis N uptake to total Nup	EVI	12	0.22	***	0.15	***	0.05		06/25	07/08	07/11					0.11	0.07			0.23	0.24	
	N harvest index	RVSI	14	0.22	***	0.14	***	0.28	**	07/07	07/08	07/11											
	Total N translocation [kg ha^–1^]	MND_750_705	13	0.42	***	0.11	**	0.21	**	06/25	05/18	07/11							0.18				62
	Leaves N translocation [kg ha^–1^]	DD	12	0.42	***	0.30	***	0.26	**	06/25	06/23	07/11				0.12	0.25	0.17	0.21	30	26	34	32
	Culms N translocation [kg ha^–1^]	RVSI	11	0.26	***	0.14	*	0.08		06/25	07/19	07/01											
	Spikes N translocation [kg ha^–1^]	NDVI4	11	0.14	***	0.28	**	0.06		06/25	06/14	05/17											
	N translocation efficiency	MND_750_705	12	0.28	***	0.26	***	0.25	**	07/07	07/19	07/11											
	Post-anthesis N uptake [kg ha^–1^]	DD	15	0.08	*	0.14	***	0.10		07/16	07/08	06/08											
other	Flowering (days in June)	MTCI	13	0.32	***	0.54	***	0.63	***	07/16	07/08	07/05				0.27	0.26	0.15	0.24	5.8	2.8	2.4	2.4

See **Figure 2** for WMMRS of all index–trait combinations. For WMMRS-based best indices, best measurements dates are reported. Using milk ripeness measurements, WMMRS-indices, NDVI2, REIP, and PLSR were calibrated in each year and validated in each other year, resulting into six validation datasets per trait. Validation R² and RMSE are reported as averaged from these six validation cases if the prediction for at least four validation cases was significant (p < 0.05). Refer to **Supplementary Table 2** for individual validation results and to **Supplementary Table 3** for calibration results.

#### Direct DM Traits

**Figure 3:**
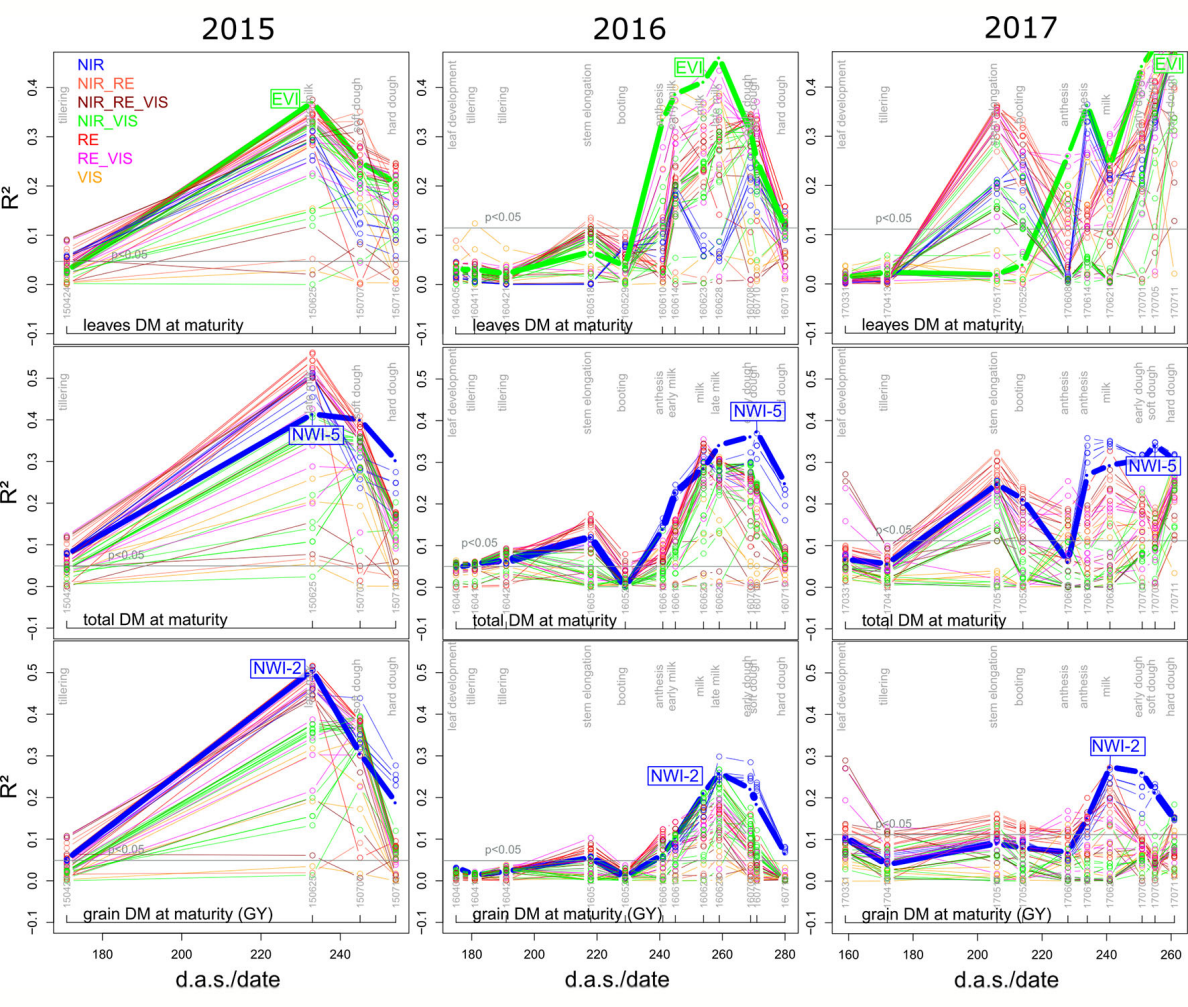
Seasonal coefficients of determination (R²) of selected direct DM traits in the three years for the tested 48 SVI. Index lines are colored according to the included spectral regions (**Supplementary Figure 1**; **Table 3**). Horizontal lines indicate the significance thresholds (p < 0.05), differing between years due to the differing number of data points. Thick lines indicate R² values of the labeled rank-based best index per trait (**Figure 2**; **Table 4**).

NIR indices showed a clear advantage for total DM at maturity (Mat; WMMRS > 12; average of all indices = 9; **Figure 2**) and for grain DM (GY; WMMRS > 14; **Figure 2**), but performed below-average for DM of leaves both at anthesis and maturity—traits RE-based indices and the EVI (WWMRS > 12) were mostly superior for (**Table 4**; **Figure 3**). Noticeably, among the large group of NIR/VIS indices (n = 15), only few indices reached superior WMMRS values. Total DM at anthesis was better estimated in 2015 (WMMRS-index: R760_R730: max. R² = 0.33***; *: *p* < 0.05; **: *p* < 0.01; ***: *p* < 0.001) and 2017 (max. R² = 0.33***) than in 2016 (max. R² = 0.11***; **Table 4**). A pronounced depression with low R² values is visible for booting in 2016 and for anthesis in 2017 for most traits (**Figure 3**). Among plant organs at anthesis, DM of leaves was best detected with slightly higher (2015 and 2016) or clearly higher (2017: R² = 0.44; EVI) *R*² values, as was found for total DM at anthesis (**Table 4**). While in 2015, indices with RE bands or only NIR bands performed similarly well during milk ripeness for DM traits (**Figure 3**), all indices with only NIR bands (blue lines) outperformed the other groups on most dates in 2016 and 2017 for total DM and GY. In all years, significant (*p* < 0.005) relationships were found for GY (grain DM at maturity; **Figure 3**) although the best R² values of the WMMRS-index NWI-2 (R² = 0.51, 0.26, 0.27) were lower in 2016 and 2017 than those found for total DM at maturity (NWI-5; R² = 0.41, 0.37, 0.34). For both traits, relationships peaked in all years at milk ripeness or early dough ripeness, and the water-related NIR indices (blue lines) excelled the other groups during grain filling and were more consistent over time. In all years, R² values of the related water band indices WBI and NWI-1 were almost identical during grain filling (**Supplementary Figure 7**). The NIR/RE indices were generally the second best group but failed at the dough stages.

#### N Concentration Traits

Weak relationships were found for all NC traits, especially at anthesis, and R² values of the WMMRS-indices (**Table 4**; **Figure 1**) differed more from the maximum relationships than for other trait groups. The R² values found from the WMMRS-index for grain NC were weak (max. R² = 0.08*) although other SVIs performed clearly better in individual years (**Supplementary Figure 4**). Relationships with maturity NC traits were closer than with anthesis NC (maximum R² of WMMRS-indices for culms: 0.26*** in 2015, 0.18* in 2016 and 0.22** in 2017; for leaves: 0.32***, 0.42** and 0.21**; **Table 4**).

#### N Uptake Traits

N uptake traits were best assessed in 2017, while the relationships were often weaker than for DM traits in the previous years (**Table 4**).

**Figure 4:**
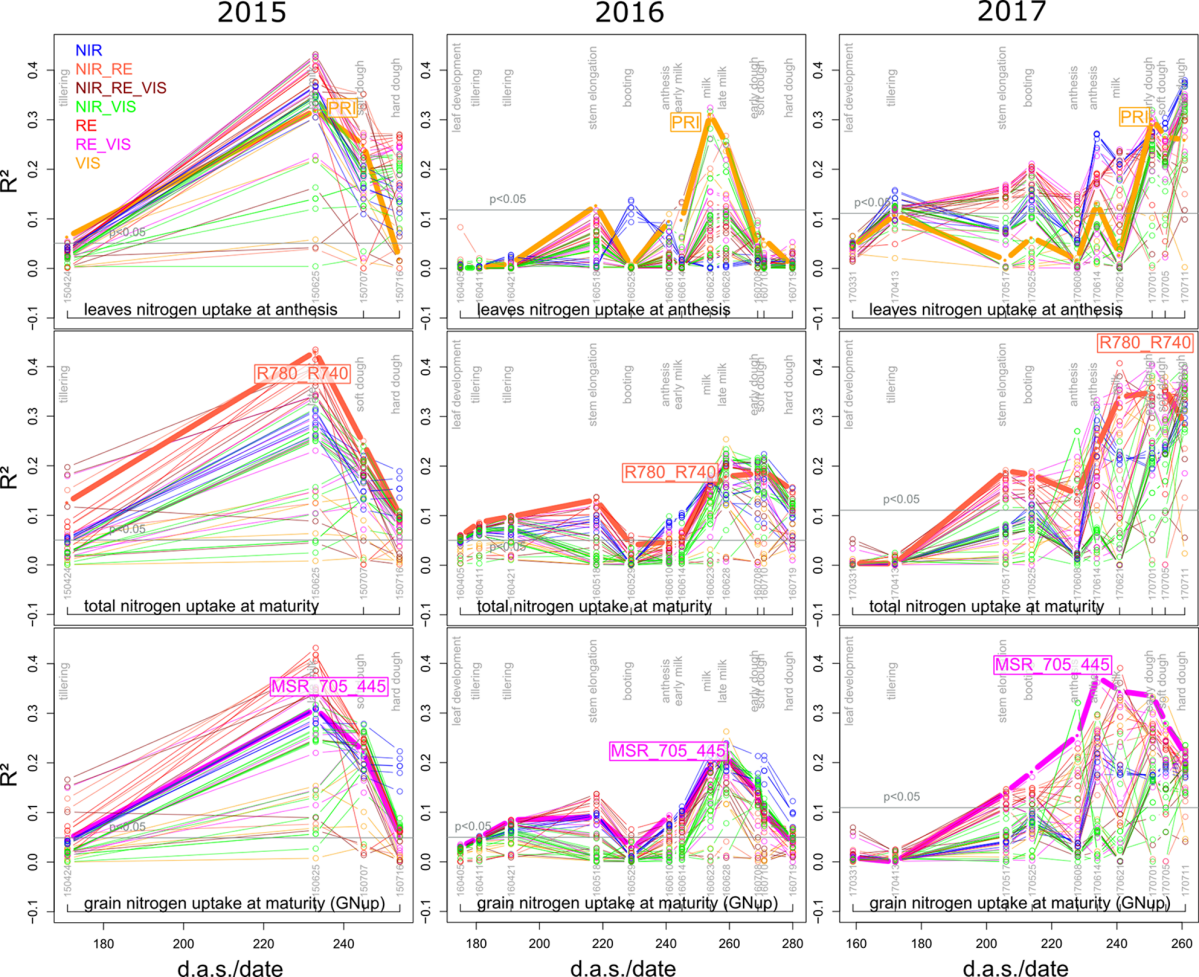
Seasonal coefficients of determination (R²) for selected N uptake (Nup) traits. See the legend of **Figure 3** for details.

**Figure 5:**
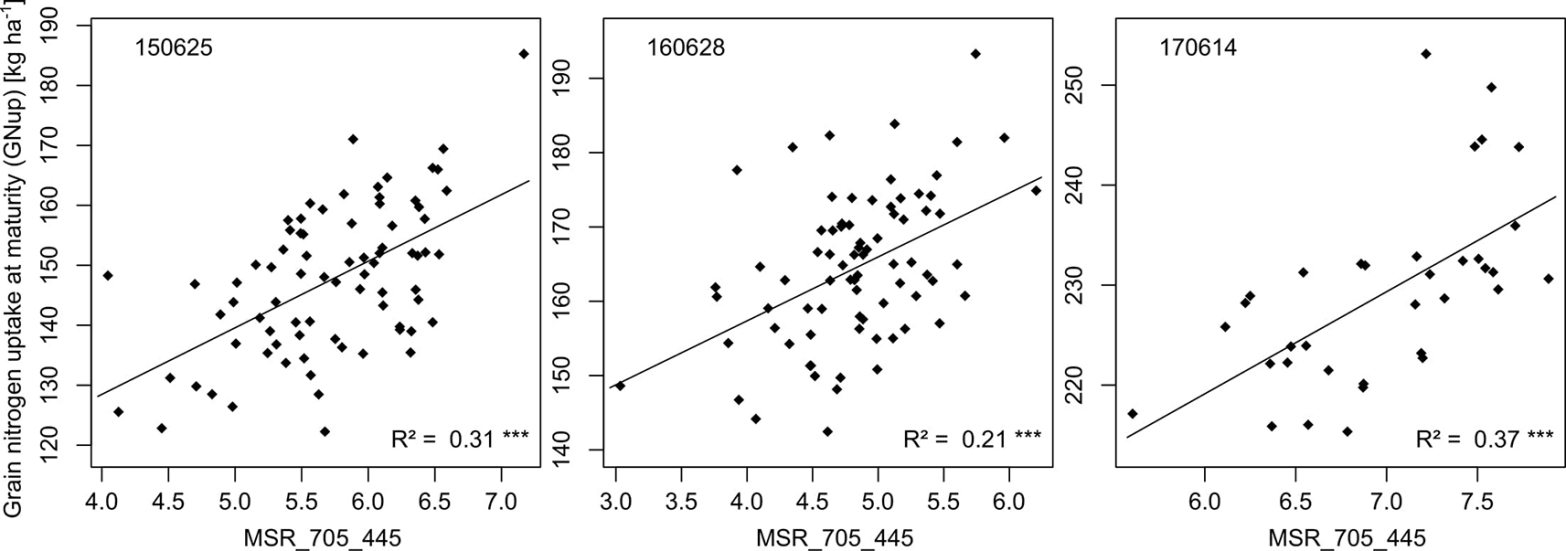
Relationships of the rank-based best index MSR_705_445 (R750-R445)/(R705-R445) with GNup on the most suitable measurements dates.

Leaf and total Nup were better estimated than the Nup of other organs. Both in 2015 and 2017, Nup of leaves at anthesis was best detected from indices of the groups of the RE/VIS, NIR/RE whereas the PRI was identified as best WMMRS index (**Figures 4** and **5**). As for total DM and grain DM, similar R² curves were observed for total Nup (WMMRS-index R780_740) and GNup (MSR_705_445; **Figure 4**), but R² values remained higher during dough ripeness for total Nup. For both traits, the group of NIR/RE indices stood out from the others during milk ripeness notably in 2015 and 2017. The detection of the vegetative Nup differed more between years than for DM. Notably, maturity leaf Nup was best detected in 2015 (WMMRS-index R787_R765: R² = 0.28***; **Table 4**), whereas culm Nup was best detected in 2017 (WMMRS-index NDRE_770_750: R² = 0.38***). Straw Nup is an indicator for the remaining, non-harvested Nup. It was weaker and similarly estimated than total Nup in 2015 and the other years, respectively (**Table 4**).

#### Derived DM Traits

Among the three yield components spike density, thousand kernel weight and grain number per spike, no consistent relationships were found with SVIs (**Table 4**). For the DM translocation (DMT; R² = 0.20***) and its efficiency (DMTEff; R² = 0.19***), moderate R² values were found only in 2016. For post-anthesis assimilation (PAA), the WMMRS-index NWI-5 revealed useful relationships during dough ripeness in 2015 (max. R² = 0.23***) and 2016 (R² = 0.34***), but not in 2017. In contrast, the harvest index (HI) was spectrally detected only in 2017 from NIR indices from anthesis on (R² = 0.30***). The total N utilization efficiency (NutEff_total) was best detected by the NWI-2, however with substantially different seasonal curves in the three years (not shown). For grain N utilization efficiency (NutEff_grain), the WMMRS-index EVI provided moderate relationships (R² = 0.21***, 0.14***, 0.21*** in 2015, 2016, and 2017 respectively), which however turned from positive sign in 2015 to negative in the other years (**Supplementary Figure 6**).

#### Derived N Traits

As for the DM harvest index, the best relationships for the N harvest index (NHI) were found in 2017, but just like for NutEff_grain, the direction of the relationship was not consistent (**Supplementary Figure 6**). In 2015 (WMMRS-index MND_750_705: R² = 0.42***) and 2017 (0.21***), N translocation (NT) was better detected than DMT. On the organ-level, NT of leaves was detected best (DD: R² = 0.42***, 0.30***, 0.26***). Unlike post-anthesis N uptake and its contribution to total Nup, and in contrast to DMTEff, N translocation efficiency (NTEff) also yielded moderate relationships, but the direction of the regression line was not consistent (**Table 4**; **Supplementary Figure 6**; **Supplementary Figure 8**). Relations for NTEff peaked later at dough ripeness than for NT. With NT being in close relationship to total Nup at anthesis (r > 0.93 in all years; not shown), the seasonal R² values were similar as for for both traits (**Supplementary Figure 8**).

### Validation of Index and PLSR Models

**Figure 6:**
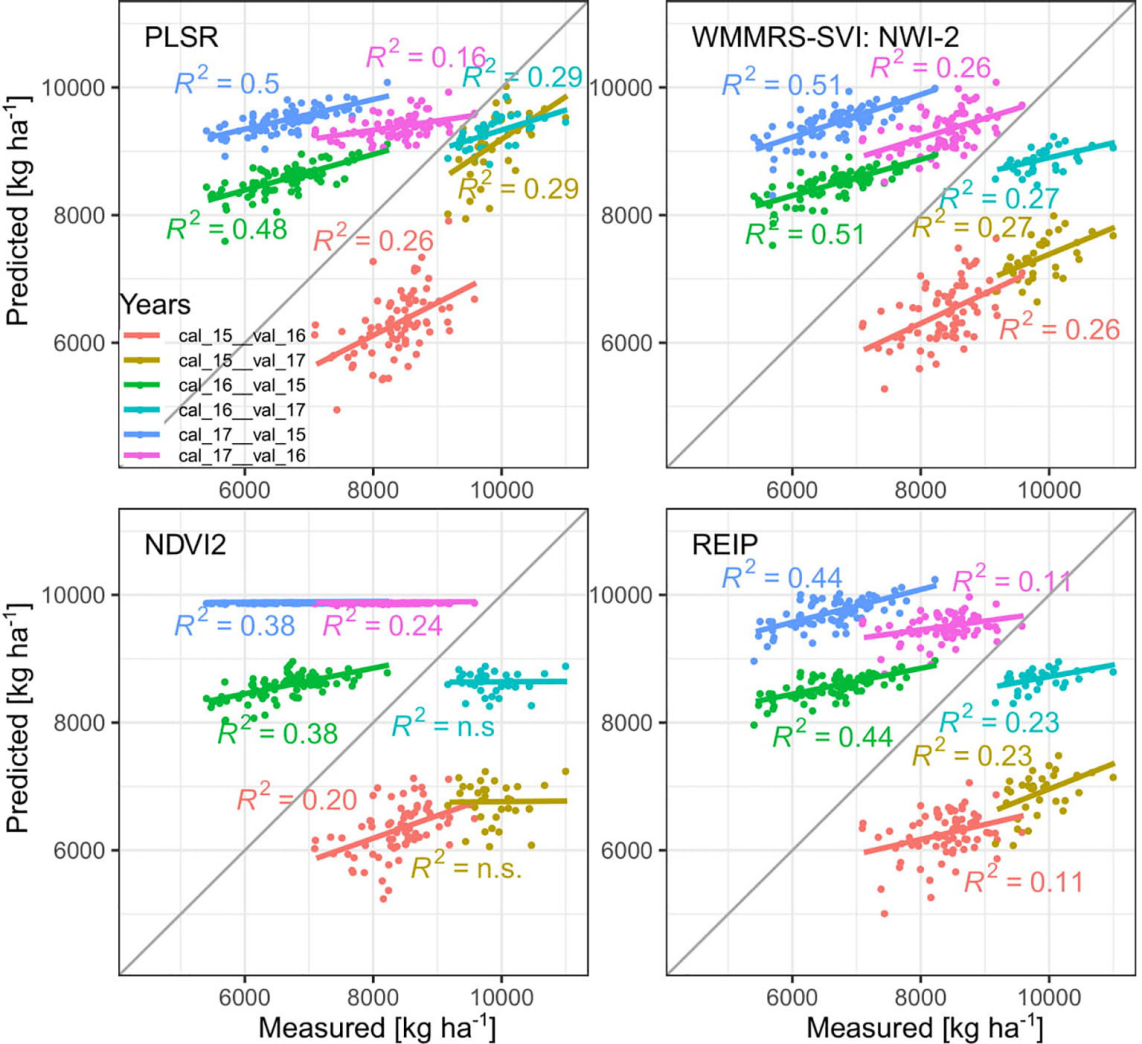
Test set validation results (*p* < 0.05) across years for GY for PLSR and index models. *Cal* and *val* indicate years of calibration and validation, respectively: 2015 (*15*), 2016 (*16*) and 2017 (*17*).

PLSR models were compared to the WMMRS-based selected index, the NDVI2 and the REIP. Due to the year-specific shifts in the spectral data and the differing seasonality, GY was substantially overestimated in 2015 (**Figure 6**). In 2017, GY predicted from PLSR models was relatively close at the 1:1 line whereas the index models resulted in substantial underestimations and low slope values. Models calibrated in 2017 overestimated GY in the other years, whereas models calibrated in 2016 over- and underestimated GY in 2015 and 2017, respectively. For GY, the WMMRS-index NWI-2 achieved similar R² values of validation but on average slightly higher RMSE values (RMSE = 1891 kg ha^−1^; **Table 4**; **Supplementary Table 2**) than the PLSR (RMSE = 1609 kg ha^−1^), while R² values were higher and RMSE values lower than from the NDVI and REIP models in all cases. Refer to **Supplementary Table 2** for all validation results and to **Supplementary Table 3** for calibration results.

For GNup, the advantage of the WMMRS-index MSR_705_445 (average R² from the six test cases = 0.29; average RMSE = 51 kg N ha^−1^) was relatively stronger with respect to the NDVI (R² = 0.20; RMSE = 56 kg N ha^−1^) but less evident with respect to the REIP (R² = 0.30; RMSE = 52 kg N ha^−1^, **Table 4**). PLSR reached lower average prediction errors (RMSE = 33 kg N ha^−1^) but not higher R² values (R² = 0.27). Compared for the averaged validation results (n = 6; **Table 4**), the WMMRS-indices achieved higher *R*² values and lower RMSE values than the PLSR models for 29 and 22 of the investigated 45 traits, for 32 and 27 traits compared to the REIP, and for 41 and 35 traits compared to the NDVI, respectively. The strongest improvement over the PLSR models was found for leaf DM at anthesis and maturity (ΔR² = +0.09 and +0.11, respectively; **Table 4**), leaf NC at maturity (ΔR² = +0.10) as well as total and leaf NT (ΔR² = +0.11 and +0.13). In contrast, PLSR was superior notably for total DM at maturity (ΔR² = +0.07), harvest index (ΔR² = +0.09) and several traits of Nup at maturity.

**Figure 7:**
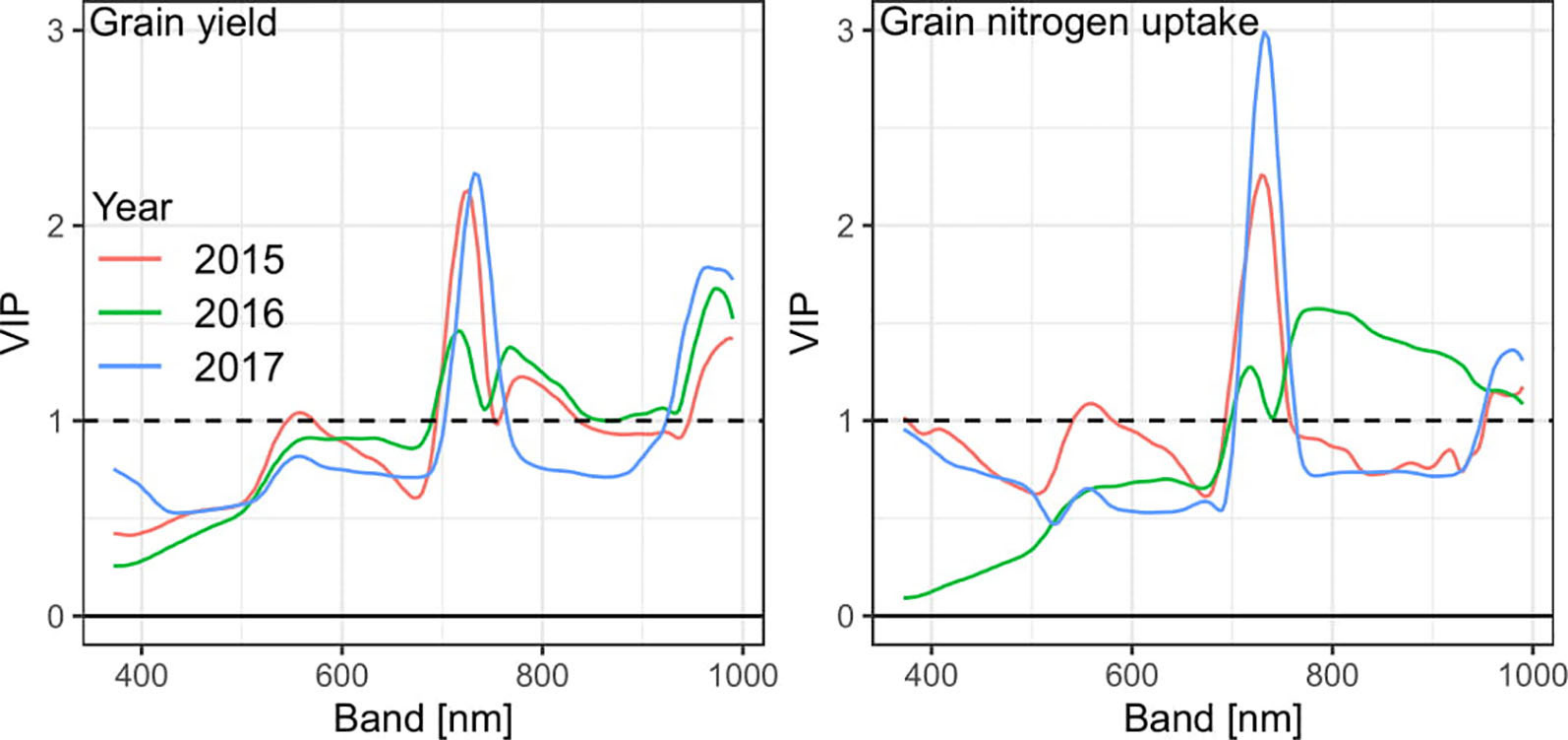
Variable Importance in Projection (VIP) of PLSR models for grain yield and grain nitrogen uptake. See **Supplementary Figure 11** for VIP values of all traits.

In addition to optimized PLSR models on derivated spectra, PLSR models were fitted on non-derivated spectra due to the shift through derivation for identifying influential wavebands. For GY, the RE region and the water band beyond about 950 nm showed highest Variable Importance in Projection (VIP) values (VIP > 1; **Figure 7**), whereas the VIS range was not particularly relevant. A similar pattern was observed for GNup, yet with a higher importance of the RE and a weaker peak at the water band. However, no pronounced RE-peak was observed for GNup in 2016.

## Discussion

The findings corroborate the possibility of early estimation of GY and GNup in optimum growth stages. Substantial variation in most traits was identified (Prey et al., 2019b), and many traits were significant for explaining plant breeders' main target traits GY, GNup, and grain N concentration (GNC), or are of direct interest. Sufficient heritability (H²) is essential for using SVIs as indirect selection tool. Increasing H² values between the vegetative and grain filling phases are in line with Frels et al. (2018) and may be due to a stronger genetic determination of the senescence process compared to the vegetative growth. The lower H² values in 2015 may be due to the only two replicates in that year, whereas the overall higher values than those found in a nearby experiment (Becker and Schmidhalter, 2017) may be associated with the larger plot size in the present experiment. The lower H² of NIR/VIS indices compared to the water (NIR) indices is in line with Babar et al. (2007) and Becker and Schmidhalter (2017).

### Optimum Growth Stages

More measurement dates than in most previous studies were tested in order to identify reliable growth stages. Overall, the grain filling phase was found to be relatively most suitable for all traits, including “post-dictions” of traits related to the anthesis canopy status. The suitability of the milk ripeness stage is in line with previous results (Freeman et al., 2003; Babar et al. 2006a; Gutierrez et al. 2010a; Christopher et al., 2014; Zhang et al., 2019). In contrast, under conditions of drought/heat-induced rapid senescence, post-anthesis assimilation is reduced and early flowering and the translocation of vegetative DM may be an escape-strategy rather than the stay-green trait (Van Herwaarden et al., 1998; Inoue et al., 2004). The canopy status at anthesis may then be more indicative than under prolonged maturation, possibly explaining the relatively better relationships of earlier dates in drier environments (Babar et al. 2006a; Prasad et al. 2007b; Becker and Schmidhalter, 2017), and the contrasting late R²-peaks in 2017, the year with the most favorable ripening conditions. The weak relationships at heading-anthesis are in line with the sensitivity of the spectral signal to the ear emergence (Pimstein et al., 2009).

### The Potential of Early Estimation of DM and GY

GY can only be predicted indirectly from spectral readings, which dominantly detect the leaf area, vegetative biomass, chlorophyll, and senescence status (Jacquemoud et al., 2009). The interrelationships of the traits contributing to GY indicated that the major fraction (56–69% in the three years) of GY was formed post-anthesis (Prey et al., 2019b). Moderate correlations were found between GY and total anthesis DM (r = 0.35, 0.43, 0.57***; not shown) in all three years, as well as with most organ-level DM traits at anthesis. In contrast to DM translocation and its efficiency, post-anthesis assimilation correlated with GY (r = 0.71***, 0.42**, and 0.69***) in all three years, explaining the better spectral relationships during grain filling.

The lower R² values in 2016 and 2017 indicate saturation of the spectral signal in dense canopies (Prasad et al., 2007b; Pavuluri et al., 2015; Frels et al., 2018). In the present dataset, the only moderate relationships in 2016 and 2017 still enable to “half” the population without losing the best-yielding genotypes—a “culling tool” strategy that would be relevant to plant breeders (Garriga et al., 2017; Frels et al., 2018) aiming at a visual evaluation of only relevant genotypes or even non-harvesting the others. The relationships were in the same range or closer than in similar studies (Pavuluri et al., 2015; Frels et al., 2018), even though the levels of DM and GY were substantially higher in the present study. With regard to plant organs, the best assessment of leaves is in line with Barmeier and Schmidhalter (2017), which was ascribed to the nadir position of the sensor, since leaves dominate the spectral signal.

#### Water Band and NIR/VIS Indices for DM and GY

In all years, the water band indices were among the best indices for GY and mostly for total DM but performed less well than most RE-based and NIR/VIS indices for leaf DM. It may be possible that the reflection in the water absorption band is influenced by the water, which is mainly located in culms and—with ongoing grain filling—in kernels, whereas the leaves' appearance dominantly impact the VIS and NIR reflection outside the water absorption band (Haboudane et al., 2004). Given that water band indices ranked relatively high during the late grain filling stages, it is also conceivable that there is a better detection of senescence traits. Total DM (r = 0.90, 0.75, 0.82 in 2015, 2016 and 2017 respectively; not shown) was dominant for explaining GY, whereas the variation in the harvest index was significant (r = 0.56***) only in 2016, explaining the similar index rankings and seasonal patterns for total DM and GY. In all years, GY correlated closer with total DM than with the DM of vegetative organs (r = 0.75 in 2016 and r > 0.82 in the other years; not shown), possibly explaining that the indirect prediction of GY from indices optimized for LAI was less successful.

**Figure 8:**
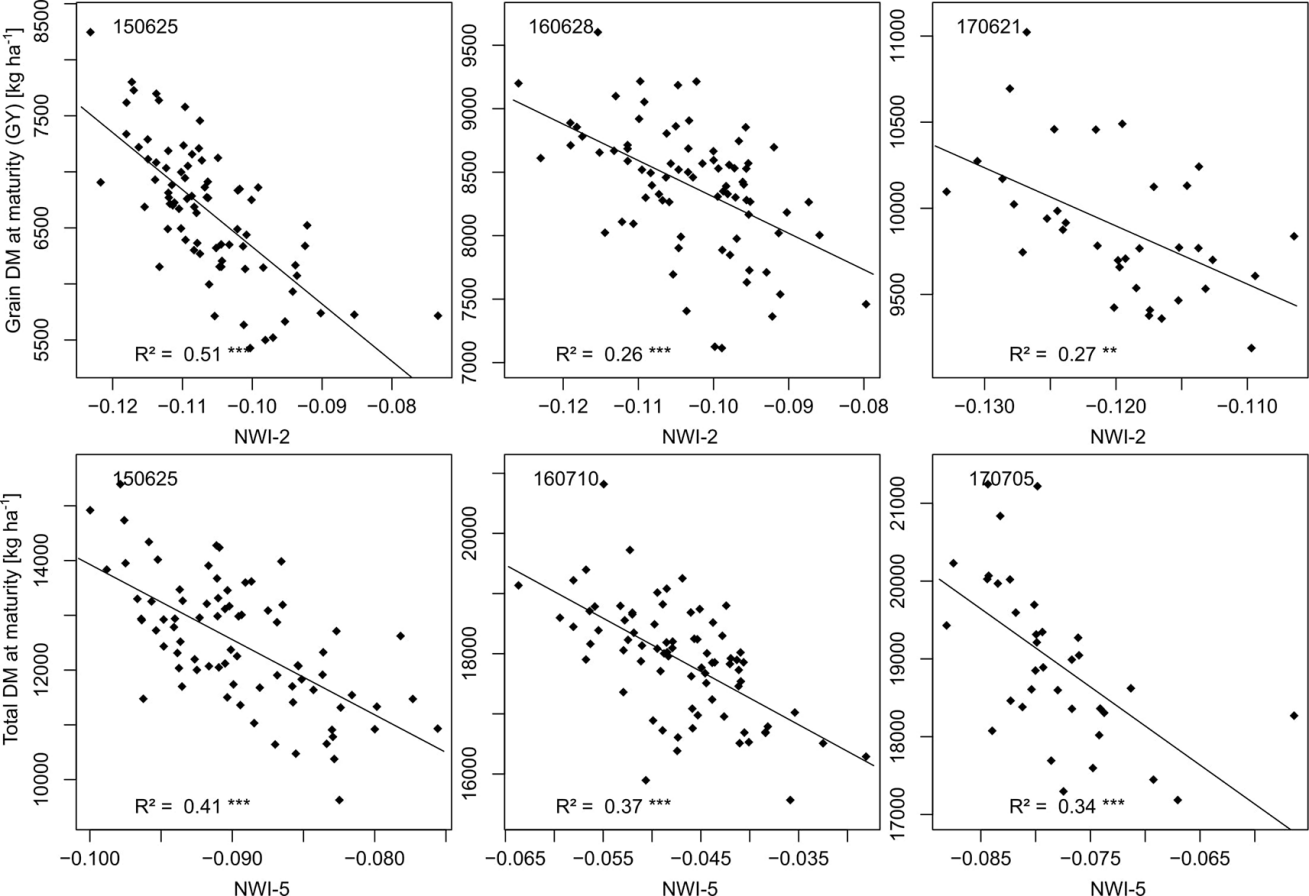
Relationships of the rank-based best indices with GY and total DM at maturity on the most suitable measurements dates (year/month/day). The index equations are (R970-R850)/(R970+R850) [NWI-2] for GY and (R970-R930)/(R970+R930) [NWI-5] for total DM at maturity.

The constant direction (**Figure 8**) of the relationships indicates that genotypes keeping canopy water later in the season also reached higher DM formation (Gutierrez et al. 2010a). This “stay-moist” trait was relatively better detected than the stay-green trait, especially in 2017, as seen from the poor performance of the VIS and NIR/VIS indices this year. In addition, water band indices were reported to be less prone to saturation than the NDVI (Sims and Gamon, 2003), corresponding to their stronger relative advantage in the highest-yielding year, 2017. The lower ranking of NIR/VIS indices optimized for LAI (EVI; MCARI1, MCARI2, MTVI2) for GY suggests that structural information that they are able to detect is less relevant for GY than the canopy water status. The present breeding population was morphologically and phenologically diverse—characteristics known to influence the spectral signal (Gutierrez et al., 2015) without direct influence on GY (Prey et al., 2019b). Among NIR/VIS and VIS indices, only the EVI ranked among the best indices for leaf DM traits, but failed for GY. It was reported to saturate less for canopies beyond NDVI values of about 0.80 (Huete et al., 2002), which were clearly exceeded from tillering to milk ripeness. The group of VIS indices ranked clearly below the other groups. Only the VARIgreen reached similar rankings as the NIR/VIS indices, as previously found for DM traits (Erdle et al., 2011).

#### Normalized Difference Versus Simple Ratio Equations

For three pairs of spectral bands, a normalized difference index and a simple ratio version were included each, namely WBI and NWI-1, GNDVI and R780_R550, as well as NDVI2 and R780_R670. For most direct and indirect DM traits, the R780_R550 (on average WMMRS Δ +0.2) and the R780_R670 (on average Δ +0.2), ranked slightly higher than the normalized difference index versions, confirming previous results (Nguy-Robertson et al., 2012; Yu et al., 2012; Barmeier and Schmidhalter, 2017).

#### RE Indices for DM and GY

Most RE-indices ranked higher than the NIR/VIS indices for most DM traits. The advantage of using wavelengths at the RE, was attributed to increased sensitivities in dense canopies (Nguy-Robertson et al., 2012; Prey and Schmidhalter, 2019a). The RE-indices were also suggested for GY (Pavuluri et al., 2015; Barmeier et al., 2017; Frels et al., 2018). For most dates, the red band used by many NIR/VIS indices was left to the position, where the reflectance difference between plots of maximum and minimum GY was most negative (**Supplementary Figure 10**). It is possible that most NIR/RE and RE indices reached higher sensitivity by positioning their lower band in this range, whereas their NIR bands were similarly positioned as those of the NIR/VIS indices, beyond approximately 760 nm at the “NIR-plateau” with similar reflectance differences, as supported by the influential bands in the PLSR models.

#### Derived DM Traits

No consistently useful estimations were achieved for the yield components, possibly because these traits were rarely correlated to GY (Prey et al., 2019b). The mostly lower coefficients of determination found for DMT compared to NT may be explained by the stronger variation in DMTEff than in NTEff. The HI was only well discriminated (R² = 0.30***) in 2017, the year when HI and total DM correlated negatively, thus indicating only indirect relationships through the detection of DM. Similarly, NutEff_grain showed negative relationships with the EVI greenness index in 2017 and 2016, but a positive relationship in 2015. In contrast, the regression of NutEff_total with its best index NWI-2 did not turn in direction, which is in line with the NutEff_total's positive correlations with total DM in all years. In contrast, Erdle et al. (2013a) found good relationships for the HI at milk ripeness, however for fewer cultivars. For NutEff_grain, Frels et al. (2018) found good relationships (max. R² = 0.41) already at heading in one year, but weaker relationships than in the present study in another year.

### The Estimation of N Traits

The usefulness of RE bands for N-related traits is well established and was related to the rightward-shift of the RE position with increasing N status (Guyot et al., 1988; Boochs et al., 1990; Guo et al., 2017) and—as for GY—the higher sensitivity in dense canopies (Erdle et al., 2011; Zhang et al., 2019). The higher ranking of most RE, NIR/RE, and NIR/RE/VIS indices may be associated with the placement of the lower band in the lower RE at 700–750 nm (**Supplementary Figure 10**) (Datt, 1999), whereas the RE/VIS indices use mostly similar red bands as the NIR/VIS indices. The results are in line with the Maccioni index that was suggested for GNup and total Nup efficiency (Frels et al., 2018), the R780_740 for detecting total Nup and for NUE (Pavuluri et al., 2015), the R760_R730 for spike Nup (Erdle et al., 2013a), as well as the NDRE_770_750 (Prey and Schmidhalter, 2019b) and the REIP (Prey et al., 2018) for GNup—all indices that ranked high for many Nup traits.

The similar best growth stages for predicting GNup just like for GY is in line with the coupling of both traits (r = 0.86, 0.66 and 0.64 in 2015, 2016 and 2017, respectively; not shown). The only date- and SVI-specific relationships found within years for GNC indicate that the formation of GNC was highly influenced by the year-specific growing conditions. Thus, the negative relationship (R² = 0.24***) found between the EVI index and GNC at milk ripeness in 2015 and the positive relationship between the senescence index PSRI and GNC in 2017 (R² = 0.18***) indicate that due to the GY/GNC antagonism, late canopy greenness was promoting GY (positive relationship with EVI, R² = 0.34***), but reducing GNC.

As the HI for GY, the NHI was secondary for explaining GNup (Prey et al., 2019b), and GNup was therefore closely correlated with total Nup (r > 0.93 in all years). This explains that the seasonal *R*² patterns and the index rankings were comparable, similarly as reported by Frels et al. (2018). In contrast to Frels et al. (2018), post-anthesis Nup was not sufficiently estimated (max. R² = 0.16***), even if it correlated positively with GNup. However, total N translocation, which was the dominating fraction for GNup in all years, revealed useful relationships during grain filling in 2015 and 2017 due to its close correlations with total Nup at anthesis. The weaker detection of vegetative Nup at maturity in organs and in the straw than of total and grain Nup may be due to the low absolute residual Nup, as well as the differing influence of the organ-level NTEff. The weak detection of N concentration (NC) traits at anthesis does not allow the recommendation of optimum indices. At maturity, moderate NC estimations were possible only for the vegetative organs but the indices previously optimized for leaf chlorophyll, TCARI_OSAVI (Haboudane et al., 2002; Huang et al., 2011), and MCARI (Daughtry et al., 2000), or for NC (R787_R765), ranked never among the best indices, thus indicating rather indirect relationships.

### Index Validation and PLSR

The comparison of the WMMRS-SVIs to the “reference” SVIs NDVIs and REIP in the year-to year test set validation models supports the usefulness of the seasonal rank-based SVI selection. The NDVI, which, despite its known limitations, is still widely used, was clearly outperformed for the vast majority of the traits by the REIP, the PLSR models, and the WMMRS-indices, confirming the results observed in the individual years. The relative advantage of the WMMRS-indices over the REIP was confirmed for GY and other DM traits, but was less pronounced for most N traits.

The often lacking or weak R²-improvements from PLSR models indicate that optimized selection of SVIs can compete with multivariate models and may be preferred in terms of calibration effort, the transferability to simpler, multispectral sensors and applicability by breeders. Thus, PLSR suggested substantial improvements in the calibration (**Supplementary Table 3**), which however largely dwindled in the validation (**Table 4**). While the relative discrimination will often be sufficient in phenotyping (Garriga et al., 2017), lower RMSE values of PLSR for several traits indicate a higher robustness over year- and growth stage-specific shifts in the spectral data, being in line with results on barley (Barmeier et al., 2017; Barmeier and Schmidhalter, 2017). Unlike to the latter study, the year-based calibrations in the present study were relatively more useful than pre-evaluated across-years models (not shown), but the validation results were generally weaker due to testing only on individual years' data. The influential bands in the PLSR confirm the RE and water bands to be most indicative.

## Conclusions

For most plant traits including GY and GNup, the milk ripeness stage was the most reliable under conditions of moderate terminal heat/drought or pathogen stress, whereas the relationships were more stable during dough ripeness in the year with favorable senescing conditions (2017). In contrast, phenological shifts at heading/anthesis appeared to decrease the relationship in this phase. NIR-combinations exploiting the water absorption band at 970 nm were found to be indispensable to achieve a useful discrimination in GY in dense canopies, followed by NIR/RE combinations, which mostly outperformed the NIR/VIS indices including the NDVI. For GNup, simple NIR/RE indices ranked high and clearly better than the NDVI. Relationships of indices with GY and GNup were explained by the detection of total DM and Nup, respectively, rather than by that of the relative allocation (harvest index) to the grain. The validation of the selected indices confirms the usefulness of the rank-based index selection notably for overcoming limitations of the NDVI. The PLSR did not achieve clearly higher R² values, but often lower estimation errors, thus that it should be preferred for improving prediction accuracies, whereas optimized SVIs appear sufficient for a relative discrimination of important traits. GNC was not reliably predicted. DM and N traits related to maturity canopy status were detected better than anthesis traits. The screening for useful band combinations can be used for optimizing sensor configurations. The results could also be transferred to multispectral sensors, thus improving the transfer of the evaluated methods to the application in breeding nurseries.

## Data Availability Statement

The datasets generated for this study are available on request to the corresponding author.

## Author Contributions

YH, LP, and US conceived and designed the experiments. LP performed the experiments. LP analyzed the data. LP and US wrote the paper.

## Funding

This research was funded by the DFG (German Research Foundation)-funded project SCHM 1456/6-1 and was partly supported by funds of the Federal Ministry of Food and Agriculture (BMEL) under the innovation support program for the project 28-1-B3.030-16.

## Conflict of Interest

The authors declare that the research was conducted in the absence of any commercial or financial relationships that could be construed as a potential conflict of interest.
