# Supplementary material for: High-Throughput Field Phenotyping Traits of Grain Yield Formation and Nitrogen Use Efficiency: Optimizing the Selection of Vegetation Indices and Growth Stages
Source: Front Plant Sci. 2020 Jan 17;10:1672. doi: 10.3389/fpls.2019.01672 (PMC6978771; doi:10.3389/fpls.2019.01672)
Supplement: Supplementary file 1 [file DataSheet_1.docx]

Supplementary Material

$$\left( a \right) {meanR^{2}}_{jts}= \frac{1}{n}\sum_{i=1}^{n} {R^{2}}_{{jts}_{i}} {\left( b \right) meanR^{2}}_{jt}= \frac{1}{m}\sum_{i=1}^{m} {{meanR^{2}}_{jts}}_{i}$$

$$\left( c \right) {maxR^{2}}_{jts}=\max\left( {R^{2}}_{{jts}_{1}},{R^{2}}_{{jts}_{2}},\ldots{R^{2}}_{{jts}_{n}} \right) (d){maxR^{2}}_{jt}= \frac{1}{m}\sum_{i=1}^{m} {{maxR^{2}}_{jts}}_{i}$$

$\left( e \right) {R\_mean}_{jts}= \frac{{meanR^{2}}_{jts}}{{meanR^{2}}_{jt}} \left( f \right) {R\_max}_{jts}= \frac{{maxR^{2}}_{jts}}{{maxR^{2}}_{jt}}$

$$\left( g \right) {RS\_mean}_{ts}= \sum_{i=1}^{l} {R\_mean}_{{jts}_{i}}\left( h \right) {RS\_max}_{ts}= \sum_{i=1}^{l} {R\_max}_{{jts}_{i}}$$

$$\left( i \right) {WMMRS}_{ts}=w*{RS\_mean}_{ts}+{RS\_max}_{ts}$$

Supplementary Equation 1: The calculation of trait-specific weighted mean/maximum rank sums (WMMRS) for all SVIs, incorporating the results of the three years. *meanR^2^_jts_* (a) denotes the seasonally average R²-value reached by a specific SVI *s* for a trait *t* in the year *j* where *n* denotes the number of dates in a given year; *meanR^2^_jt_* (b) denotes the average *meanR^2^_jts_* of all SVIs for a specific trait in the year *j* where *m* denotes the number of SVIs. Similarly, *maxR^2^_jts_* (c) denotes the seasonally maximum R² value reached by a specific SVI *s* for a trait *t* in the year *j*; *maxR^2^_jt_* (d) denotes the average *maxR^2^_jts_* of all SVIs for a specific trait in the year *j*. *R_mean_jts_* (e) and *R_max_jts_* (h) denote the mean- and maximum-based SVI rankings in the year *j* for trait *t*, respectively, which were summed up across years to rank sums *RS_mean_ts_* (g) and *RS_maxt_s,_* (h) respectively*,* where *l* is the number of years. The finally used WMMRS (i) are the weighted rank sums (weighting *w* = 2) of *RS_mean_ts_* and *RS_max_ts_*.

Supplementary Table 1: Heritability by indices, grouped by index groups, and measurement date (month/day).

|  |  |  |  | **2015** |  |  |  |  | **2016** |  |  |  |  |  |  |  |  |  |  |  |  |  | **2017** |  |  |  |  |
| --- | --- | --- | --- | --- | --- | --- | --- | --- | --- | --- | --- | --- | --- | --- | --- | --- | --- | --- | --- | --- | --- | --- | --- | --- | --- | --- | --- |
|  |  | **tillering** | **late milk** | **soft dough** | **hard dough** | **leaf development** | **tillering** | **tillering** | **stem elongation** | **booting** | **anthesis** | **early milk** | **milk** | **late milk** | **early dough** | **soft dough** | **hard dough** | **leaf development** | **tillering** | **stem elongation** | **booting** | **anthesis** | **anthesis** | **milk** | **early dough** | **soft dough** | **hard dough** |
| **group** | **Index** | **04/24** | **06/25** | **07/07** | **07/16** | **04/05** | **04/11** | **04/21** | **05/18** | **05/29** | **06/10** | **06/14** | **06/23** | **06/28** | **07/08** | **07/10** | **07/19** | **03/31** | **04/13** | **05/17** | **05/25** | **06/08** | **06/14** | **06/21** | **07/01** | **07/05** | **0/711** |
| **NIR** | **NWI-1** | 0.30 | 0.37 | 0.49 | 0.73 | 0.59 | 0.58 | 0.77 | 0.81 | 0.92 | 0.91 | 0.91 | 0.85 | 0.84 | 0.77 | 0.83 | 0.76 | 0.77 | 0.70 | 0.85 | 0.84 | 0.94 | 0.93 | 0.82 | 0.86 | 0.57 | 0.96 |
| **NIR** | **NWI-2** | 0.29 | 0.53 | 0.56 | 0.78 | 0.62 | 0.58 | 0.79 | 0.79 | 0.91 | 0.87 | 0.92 | 0.87 | 0.84 | 0.83 | 0.89 | 0.74 | 0.77 | 0.67 | 0.85 | 0.78 | 0.94 | 0.92 | 0.80 | 0.88 | 0.68 | 0.96 |
| **NIR** | **NWI-3** | 0.28 | 0.31 | 0.49 | 0.71 | 0.58 | 0.58 | 0.76 | 0.83 | 0.91 | 0.93 | 0.91 | 0.84 | 0.85 | 0.75 | 0.80 | 0.71 | 0.76 | 0.71 | 0.85 | 0.85 | 0.94 | 0.93 | 0.84 | 0.85 | 0.51 | 0.96 |
| **NIR** | **NWI-4** | 0.29 | 0.43 | 0.50 | 0.75 | 0.61 | 0.58 | 0.77 | 0.79 | 0.92 | 0.89 | 0.91 | 0.85 | 0.82 | 0.78 | 0.85 | 0.73 | 0.77 | 0.68 | 0.85 | 0.80 | 0.94 | 0.92 | 0.81 | 0.86 | 0.60 | 0.96 |
| **NIR** | **NWI-5** | 0.28 | 0.34 | 0.61 | 0.75 | 0.53 | 0.59 | 0.75 | 0.81 | 0.89 | 0.93 | 0.90 | 0.83 | 0.84 | 0.79 | 0.77 | 0.74 | 0.77 | 0.74 | 0.86 | 0.82 | 0.94 | 0.93 | 0.86 | 0.84 | 0.60 | 0.95 |
| **NIR** | **WBI** | 0.31 | 0.37 | 0.49 | 0.73 | 0.59 | 0.58 | 0.77 | 0.81 | 0.92 | 0.91 | 0.91 | 0.85 | 0.84 | 0.77 | 0.82 | 0.76 | 0.77 | 0.70 | 0.85 | 0.84 | 0.94 | 0.92 | 0.82 | 0.86 | 0.49 | 0.96 |
| **NIR_VIS** | **EVI** | 0.34 | 0.80 | 0.70 | 0.76 | 0.58 | 0.65 | 0.84 | 0.54 | 0.76 | 0.93 | 0.93 | 0.89 | 0.88 | 0.86 | 0.81 | 0.90 | 0.76 | 0.77 | 0.46 | 0.00 | 0.87 | 0.57 | 0.54 | 0.83 | 0.93 | 0.96 |
| **NIR_VIS** | **GNDVI** | 0.16 | 0.66 | 0.65 | 0.81 | 0.61 | 0.61 | 0.68 | 0.53 | 0.79 | 0.88 | 0.88 | 0.86 | 0.90 | 0.87 | 0.85 | 0.91 | 0.77 | 0.75 | 0.80 | 0.46 | 0.93 | 0.70 | 0.67 | 0.82 | 0.90 | 0.96 |
| **NIR_VIS** | **MCARI1** | 0.30 | 0.63 | 0.74 | 0.79 | 0.66 | 0.80 | 0.89 | 0.91 | 0.95 | 0.86 | 0.91 | 0.88 | 0.85 | 0.84 | 0.82 | 0.88 | 0.77 | 0.76 | 0.88 | 0.84 | 0.80 | 0.82 | 0.75 | 0.91 | 0.94 | 0.96 |
| **NIR_VIS** | **MCARI2** | 0.26 | 0.81 | 0.62 | 0.77 | 0.63 | 0.71 | 0.87 | 0.37 | 0.54 | 0.90 | 0.90 | 0.82 | 0.79 | 0.83 | 0.80 | 0.90 | 0.76 | 0.72 | 0.48 | 0.00 | 0.87 | 0.10 | 0.00 | 0.61 | 0.89 | 0.97 |
| **NIR_VIS** | **MSAVI** | 0.20 | 0.64 | 0.62 | 0.77 | 0.62 | 0.67 | 0.80 | 0.41 | 0.52 | 0.83 | 0.87 | 0.80 | 0.84 | 0.83 | 0.79 | 0.90 | 0.76 | 0.73 | 0.54 | 0.00 | 0.92 | 0.25 | 0.14 | 0.70 | 0.88 | 0.97 |
| **NIR_VIS** | **MSR_670** | 0.36 | 0.65 | 0.67 | 0.76 | 0.56 | 0.59 | 0.76 | 0.53 | 0.69 | 0.83 | 0.86 | 0.83 | 0.86 | 0.85 | 0.80 | 0.89 | 0.77 | 0.80 | 0.54 | 0.00 | 0.93 | 0.34 | 0.28 | 0.67 | 0.87 | 0.95 |
| **NIR_VIS** | **MTVI2** | 0.26 | 0.81 | 0.62 | 0.77 | 0.63 | 0.71 | 0.88 | 0.37 | 0.54 | 0.90 | 0.90 | 0.82 | 0.79 | 0.83 | 0.80 | 0.90 | 0.76 | 0.72 | 0.48 | 0.00 | 0.87 | 0.10 | 0.00 | 0.61 | 0.89 | 0.97 |
| **NIR_VIS** | **NDVI1** | 0.26 | 0.63 | 0.62 | 0.76 | 0.61 | 0.66 | 0.80 | 0.41 | 0.54 | 0.83 | 0.86 | 0.80 | 0.84 | 0.84 | 0.79 | 0.90 | 0.77 | 0.75 | 0.54 | 0.00 | 0.92 | 0.27 | 0.15 | 0.68 | 0.88 | 0.96 |
| **NIR_VIS** | **NDVI2** | 0.26 | 0.65 | 0.63 | 0.77 | 0.61 | 0.66 | 0.80 | 0.42 | 0.53 | 0.84 | 0.87 | 0.81 | 0.84 | 0.85 | 0.80 | 0.90 | 0.77 | 0.75 | 0.54 | 0.00 | 0.92 | 0.26 | 0.15 | 0.70 | 0.89 | 0.96 |
| **NIR_VIS** | **NDVI3** | 0.26 | 0.63 | 0.62 | 0.77 | 0.61 | 0.66 | 0.79 | 0.41 | 0.54 | 0.82 | 0.85 | 0.79 | 0.84 | 0.83 | 0.79 | 0.91 | 0.76 | 0.75 | 0.54 | 0.00 | 0.92 | 0.27 | 0.15 | 0.68 | 0.88 | 0.96 |
| **NIR_VIS** | **OSAVI** | 0.26 | 0.64 | 0.63 | 0.76 | 0.61 | 0.66 | 0.80 | 0.43 | 0.53 | 0.83 | 0.87 | 0.80 | 0.84 | 0.84 | 0.80 | 0.90 | 0.77 | 0.75 | 0.54 | 0.00 | 0.92 | 0.26 | 0.15 | 0.70 | 0.89 | 0.96 |
| **NIR_VIS** | **PSSR** | 0.34 | 0.80 | 0.67 | 0.84 | 0.55 | 0.54 | 0.70 | 0.61 | 0.87 | 0.92 | 0.93 | 0.89 | 0.92 | 0.84 | 0.80 | 0.89 | 0.78 | 0.81 | 0.71 | 0.32 | 0.94 | 0.71 | 0.73 | 0.75 | 0.85 | 0.95 |
| **NIR_VIS** | **R780_R550** | 0.26 | 0.65 | 0.68 | 0.80 | 0.56 | 0.53 | 0.63 | 0.56 | 0.82 | 0.86 | 0.87 | 0.85 | 0.90 | 0.86 | 0.83 | 0.90 | 0.77 | 0.80 | 0.77 | 0.51 | 0.92 | 0.71 | 0.71 | 0.81 | 0.89 | 0.95 |
| **NIR_VIS** | **R780_R670** | 0.40 | 0.65 | 0.71 | 0.75 | 0.52 | 0.53 | 0.73 | 0.56 | 0.73 | 0.83 | 0.86 | 0.84 | 0.87 | 0.85 | 0.79 | 0.89 | 0.77 | 0.82 | 0.54 | 0.06 | 0.94 | 0.37 | 0.33 | 0.67 | 0.85 | 0.94 |
| **NIR_VIS** | **WDRVI** | 0.37 | 0.65 | 0.67 | 0.76 | 0.57 | 0.60 | 0.78 | 0.47 | 0.61 | 0.83 | 0.86 | 0.82 | 0.86 | 0.85 | 0.80 | 0.89 | 0.77 | 0.80 | 0.54 | 0.00 | 0.93 | 0.29 | 0.22 | 0.68 | 0.88 | 0.95 |
| **VIS** | **ARI** | 0.65 | 0.87 | 0.88 | 0.89 | 0.68 | 0.71 | 0.97 | 0.92 | 0.91 | 0.95 | 0.96 | 0.95 | 0.93 | 0.94 | 0.97 | 0.94 | 0.65 | 0.82 | 0.90 | 0.81 | 0.96 | 0.94 | 0.96 | 0.92 | 0.90 | 0.95 |
| **VIS** | **BGI** | 0.56 | 0.97 | 0.89 | 0.97 | 0.67 | 0.74 | 0.86 | 0.74 | 0.97 | 0.99 | 0.99 | 0.99 | 0.99 | 0.91 | 0.90 | 0.94 | 0.81 | 0.83 | 0.79 | 0.52 | 0.98 | 0.98 | 0.98 | 0.97 | 0.95 | 0.96 |
| **VIS** | **BRI** | 0.49 | 0.90 | 0.91 | 0.94 | 0.72 | 0.76 | 0.91 | 0.82 | 0.93 | 0.97 | 0.97 | 0.97 | 0.97 | 0.98 | 0.99 | 0.93 | 0.55 | 0.80 | 0.72 | 0.78 | 0.96 | 0.89 | 0.93 | 0.98 | 0.98 | 0.98 |
| **VIS** | **PRI** | 0.41 | 0.82 | 0.87 | 0.97 | 0.58 | 0.60 | 0.85 | 0.59 | 0.54 | 0.88 | 0.91 | 0.94 | 0.95 | 0.96 | 0.98 | 0.91 | 0.75 | 0.80 | 0.44 | 0.07 | 0.78 | 0.36 | 0.36 | 0.87 | 0.96 | 0.96 |
| **VIS** | **VARIgreen** | 0.51 | 0.73 | 0.70 | 0.81 | 0.57 | 0.66 | 0.86 | 0.51 | 0.49 | 0.78 | 0.81 | 0.83 | 0.84 | 0.88 | 0.86 | 0.89 | 0.77 | 0.81 | 0.43 | 0.00 | 0.74 | 0.06 | 0.00 | 0.61 | 0.89 | 0.96 |
| **NIR_RE** | **R787_R765** | 0.77 | 0.73 | 0.77 | 0.80 | 0.58 | 0.66 | 0.76 | 0.79 | 0.85 | 0.89 | 0.74 | 0.92 | 0.96 | 0.93 | 0.96 | 0.89 | 0.72 | 0.78 | 0.86 | 0.84 | 0.95 | 0.96 | 0.94 | 0.84 | 0.90 | 0.94 |
| **NIR_RE** | **NDRE** | 0.15 | 0.61 | 0.68 | 0.74 | 0.58 | 0.54 | 0.60 | 0.60 | 0.82 | 0.88 | 0.88 | 0.86 | 0.90 | 0.88 | 0.83 | 0.91 | 0.76 | 0.75 | 0.82 | 0.76 | 0.93 | 0.85 | 0.84 | 0.91 | 0.94 | 0.95 |
| **NIR_RE** | **NDRE_770_750** | 0.17 | 0.54 | 0.68 | 0.75 | 0.53 | 0.45 | 0.49 | 0.57 | 0.88 | 0.90 | 0.88 | 0.89 | 0.90 | 0.89 | 0.87 | 0.91 | 0.73 | 0.77 | 0.80 | 0.87 | 0.93 | 0.93 | 0.92 | 0.93 | 0.94 | 0.94 |
| **NIR_RE** | **R780_R740** | 0.16 | 0.56 | 0.68 | 0.73 | 0.55 | 0.46 | 0.50 | 0.59 | 0.87 | 0.90 | 0.88 | 0.88 | 0.90 | 0.88 | 0.85 | 0.91 | 0.75 | 0.77 | 0.81 | 0.85 | 0.92 | 0.92 | 0.91 | 0.93 | 0.94 | 0.94 |
| **NIR_RE_VIS** | **Maccioni** | 0.12 | 0.70 | 0.76 | 0.73 | 0.60 | 0.51 | 0.56 | 0.67 | 0.91 | 0.92 | 0.92 | 0.91 | 0.93 | 0.92 | 0.91 | 0.89 | 0.78 | 0.71 | 0.87 | 0.93 | 0.93 | 0.95 | 0.95 | 0.97 | 0.97 | 0.94 |
| **NIR_RE_VIS** | **REIP** | 0.15 | 0.61 | 0.76 | 0.75 | 0.59 | 0.47 | 0.51 | 0.58 | 0.91 | 0.92 | 0.90 | 0.91 | 0.92 | 0.93 | 0.93 | 0.89 | 0.74 | 0.71 | 0.83 | 0.91 | 0.93 | 0.95 | 0.95 | 0.96 | 0.96 | 0.93 |
| **NIR_RE_VIS** | **TCARI_OSAVI** | 0.18 | 0.89 | 0.83 | 0.80 | 0.62 | 0.49 | 0.69 | 0.96 | 0.97 | 0.95 | 0.95 | 0.95 | 0.96 | 0.93 | 0.96 | 0.90 | 0.70 | 0.73 | 0.95 | 0.95 | 0.97 | 0.95 | 0.96 | 0.94 | 0.94 | 0.95 |
| **NIR_RE_VIS** | **LCI** | 0.20 | 0.61 | 0.65 | 0.75 | 0.59 | 0.60 | 0.71 | 0.50 | 0.66 | 0.81 | 0.85 | 0.82 | 0.88 | 0.86 | 0.81 | 0.91 | 0.77 | 0.75 | 0.75 | 0.39 | 0.93 | 0.63 | 0.58 | 0.85 | 0.92 | 0.96 |
| **RE** | **R760_R730** | 0.17 | 0.59 | 0.70 | 0.74 | 0.56 | 0.49 | 0.56 | 0.62 | 0.85 | 0.89 | 0.89 | 0.87 | 0.90 | 0.88 | 0.83 | 0.89 | 0.77 | 0.76 | 0.81 | 0.81 | 0.92 | 0.88 | 0.87 | 0.93 | 0.95 | 0.95 |
| **RE** | **RVSI** | 0.50 | 0.57 | 0.76 | 0.80 | 0.78 | 0.73 | 0.78 | 0.58 | 0.89 | 0.87 | 0.89 | 0.90 | 0.92 | 0.82 | 0.84 | 0.85 | 0.78 | 0.77 | 0.89 | 0.94 | 0.92 | 0.94 | 0.95 | 0.94 | 0.93 | 0.96 |
| **RE** | **HVI** | 0.31 | 0.63 | 0.71 | 0.76 | 0.56 | 0.55 | 0.72 | 0.56 | 0.66 | 0.79 | 0.84 | 0.84 | 0.88 | 0.87 | 0.81 | 0.89 | 0.78 | 0.79 | 0.71 | 0.38 | 0.91 | 0.54 | 0.55 | 0.83 | 0.92 | 0.95 |
| **RE** | **VOG1** | 0.20 | 0.63 | 0.70 | 0.76 | 0.58 | 0.54 | 0.65 | 0.60 | 0.78 | 0.85 | 0.87 | 0.85 | 0.89 | 0.88 | 0.82 | 0.89 | 0.77 | 0.76 | 0.81 | 0.68 | 0.92 | 0.78 | 0.76 | 0.89 | 0.94 | 0.95 |
| **RE** | **VOG2** | 0.19 | 0.60 | 0.71 | 0.74 | 0.56 | 0.50 | 0.59 | 0.62 | 0.83 | 0.88 | 0.88 | 0.86 | 0.89 | 0.88 | 0.83 | 0.89 | 0.77 | 0.77 | 0.81 | 0.77 | 0.92 | 0.85 | 0.84 | 0.91 | 0.94 | 0.95 |
| **RE_VIS** | **DD** | 0.15 | 0.60 | 0.76 | 0.75 | 0.61 | 0.64 | 0.80 | 0.89 | 0.90 | 0.82 | 0.90 | 0.88 | 0.88 | 0.89 | 0.86 | 0.77 | 0.79 | 0.75 | 0.77 | 0.67 | 0.41 | 0.69 | 0.80 | 0.95 | 0.96 | 0.94 |
| **RE_VIS** | **MCARI** | 0.61 | 0.92 | 0.73 | 0.80 | 0.66 | 0.72 | 0.91 | 0.76 | 0.96 | 0.97 | 0.97 | 0.96 | 0.92 | 0.83 | 0.84 | 0.87 | 0.78 | 0.83 | 0.85 | 0.70 | 0.97 | 0.85 | 0.78 | 0.75 | 0.90 | 0.96 |
| **RE_VIS** | **MND_750_705** | 0.15 | 0.73 | 0.71 | 0.77 | 0.61 | 0.60 | 0.72 | 0.61 | 0.75 | 0.90 | 0.92 | 0.90 | 0.91 | 0.88 | 0.84 | 0.90 | 0.76 | 0.72 | 0.77 | 0.62 | 0.94 | 0.80 | 0.84 | 0.94 | 0.96 | 0.96 |
| **RE_VIS** | **MSR705_445** | 0.23 | 0.69 | 0.74 | 0.76 | 0.57 | 0.54 | 0.69 | 0.62 | 0.77 | 0.90 | 0.91 | 0.90 | 0.91 | 0.89 | 0.83 | 0.89 | 0.77 | 0.76 | 0.76 | 0.64 | 0.93 | 0.80 | 0.84 | 0.92 | 0.95 | 0.95 |
| **RE_VIS** | **MTCI** | 0.14 | 0.69 | 0.75 | 0.77 | 0.58 | 0.47 | 0.56 | 0.69 | 0.91 | 0.92 | 0.92 | 0.90 | 0.92 | 0.92 | 0.88 | 0.84 | 0.77 | 0.74 | 0.86 | 0.92 | 0.92 | 0.94 | 0.94 | 0.96 | 0.96 | 0.95 |
| **RE_VIS** | **NDVI4** | 0.19 | 0.65 | 0.68 | 0.76 | 0.60 | 0.60 | 0.72 | 0.55 | 0.66 | 0.83 | 0.88 | 0.85 | 0.89 | 0.87 | 0.82 | 0.90 | 0.77 | 0.75 | 0.78 | 0.49 | 0.92 | 0.65 | 0.64 | 0.88 | 0.94 | 0.96 |
| **RE_VIS** | **PSRI** | 0.27 | 0.83 | 0.69 | 0.80 | 0.60 | 0.68 | 0.86 | 0.52 | 0.72 | 0.91 | 0.92 | 0.88 | 0.86 | 0.87 | 0.84 | 0.90 | 0.75 | 0.71 | 0.36 | 0.00 | 0.83 | 0.48 | 0.46 | 0.84 | 0.94 | 0.97 |
| **RE_VIS** | **R730_R670** | 0.43 | 0.76 | 0.70 | 0.76 | 0.54 | 0.58 | 0.78 | 0.55 | 0.75 | 0.87 | 0.89 | 0.87 | 0.87 | 0.85 | 0.79 | 0.88 | 0.78 | 0.82 | 0.49 | 0.00 | 0.95 | 0.35 | 0.30 | 0.59 | 0.83 | 0.95 |
| **RE_VIS** | **R760_R670** | 0.40 | 0.66 | 0.71 | 0.76 | 0.52 | 0.54 | 0.73 | 0.56 | 0.73 | 0.83 | 0.86 | 0.84 | 0.87 | 0.86 | 0.79 | 0.89 | 0.78 | 0.82 | 0.53 | 0.05 | 0.94 | 0.36 | 0.33 | 0.67 | 0.85 | 0.94 |

Supplementary Table 2: Validation results (only *p* < 0.05) of cross-wise test validations with PLSR, NDVI2, REIP and the WMMRS-based best trait-specific vegetation indices. Refer to Table 4 for averaged results, for the names of the WMMRS-indices and trait units. Refer to Supplementary Table 3 for calibration results.

Supplementary Table 3: Results of year-wise calibration models at milk ripeness: Coefficients of determination (R²) and root mean square error (RMSE). See Table 4 for trait units.

|  | Calibration year | Model | total DM at anthesis | total DM at maturity | grain DM at maturity | leaves DM at anthesis | spikes DM at anthesis | culms DM at anthesis | leaves DM at maturity | culms DM at maturity | chaff DM at maturity | spike density | contribution of post anthesis  assimilation to grain filling | post anthesis assimilation | DM translocation | DM translocation efficiency | harvest index | grain nitrogen utilization efficiency | total nitrogen utilization efficiency | grain number per spike | thousand kernel weight | spikes nitrogen uptake at anthesis | leaves nitrogen uptake at anthesis | culms nitrogen uptake at anthesis | total nitrogen uptake at anthesis | leaves nitrogen uptake at maturity | culms nitrogen uptake at maturity | grain nitrogen uptake at maturity | total nitrogen uptake at maturity | chaff nitrogen uptake at maturity | straw nitrogen uptake at maturity | spikes nitrogen concentration at anthesis | leaves nitrogen concentration at anthesis | culms nitrogen concentration at anthesis | leaves nitrogen concentration at maturity | culms nitrogen concentration at maturity | grain nitrogen concentration at maturity | chaff nitrogen concentration at maturity | contribution of post anthesis N  uptake to total nitrogen uptake | nitrogen harvest index | spikes nitrogen translocation | leaves nitrogen translocation | culms nitrogen translocation | total nitrogen translocation | nitrogen translocation efficiency | post anthesis nitrogen uptake | flowering_days_in_June |
| --- | --- | --- | --- | --- | --- | --- | --- | --- | --- | --- | --- | --- | --- | --- | --- | --- | --- | --- | --- | --- | --- | --- | --- | --- | --- | --- | --- | --- | --- | --- | --- | --- | --- | --- | --- | --- | --- | --- | --- | --- | --- | --- | --- | --- | --- | --- | --- |
| R² (calibration) | 15 | PLSR | 0.38 | 0.61 | 0.62 | 0.67 | 0.19 | 0.35 | 0.65 | 0.57 | 0.16 | 0.33 | 0.02 | 0.22 | 0.05 | 0.30 | 0.23 | 0.35 | 0.52 | 0.01 | 0.23 | 0.23 | 0.65 | 0.31 | 0.45 | 0.42 | 0.30 | 0.58 | 0.54 | 0.05 | 0.41 | 0.02 | 0.19 | 0.01 | 0.54 | 0.39 | 0.36 | 0.02 | 0.36 | 0.54 | 0.36 | 0.52 | 0.32 | 0.49 | 0.51 | 0.38 | 0.02 |
|  | 16 | PLSR | 0.00 | 0.49 | 0.35 | 0.44 | 0.20 | 0.02 | 0.73 | 0.48 | 0.13 | 0.27 | 0.21 | 0.34 | 0.12 | 0.24 | 0.20 | 0.15 | 0.01 | 0.34 | 0.27 | 0.02 | 0.52 | 0.08 | 0.02 | 0.72 | 0.58 | 0.27 | 0.40 | 0.08 | 0.44 | 0.24 | 0.04 | 0.15 | 0.76 | 0.76 | 0.01 | 0.01 | 0.12 | 0.41 | 0.58 | 0.53 | 0.15 | 0.01 | 0.42 | 0.14 | 0.56 |
|  | 17 | PLSR | 0.45 | 0.55 | 0.40 | 0.18 | 0.40 | 0.34 | 0.49 | 0.50 | 0.31 | 0.08 | 0.01 | 0.05 | 0.01 | 0.04 | 0.43 | 0.15 | 0.24 | 0.05 | 0.03 | 0.23 | 0.26 | 0.17 | 0.28 | 0.18 | 0.31 | 0.41 | 0.46 | 0.16 | 0.29 | 0.04 | 0.06 | 0.05 | 0.06 | 0.11 | 0.06 | 0.14 | 0.03 | 0.18 | 0.21 | 0.20 | 0.07 | 0.18 | 0.20 | 0.06 | 0.39 |
|  | 15 | NDVI2 | 0.21 | 0.37 | 0.38 | 0.34 | 0.12 | 0.15 | 0.32 | 0.23 | 0.08 | 0.15 | 0.01 | 0.15 | 0.03 | 0.01 | 0.00 | 0.11 | 0.14 | 0.01 | 0.02 | 0.13 | 0.35 | 0.16 | 0.29 | 0.02 | 0.01 | 0.25 | 0.25 | 0.03 | 0.03 | 0.00 | 0.08 | 0.00 | 0.15 | 0.14 | 0.10 | 0.01 | 0.09 | 0.02 | 0.11 | 0.37 | 0.19 | 0.32 | 0.16 | 0.00 | 0.06 |
|  | 16 | NDVI2 | 0.01 | 0.31 | 0.24 | 0.14 | 0.09 | 0.03 | 0.33 | 0.30 | 0.04 | 0.05 | 0.16 | 0.26 | 0.08 | 0.15 | 0.00 | 0.00 | 0.01 | 0.02 | 0.00 | 0.00 | 0.12 | 0.01 | 0.00 | 0.00 | 0.29 | 0.22 | 0.22 | 0.06 | 0.02 | 0.12 | 0.01 | 0.06 | 0.22 | 0.02 | 0.00 | 0.02 | 0.10 | 0.03 | 0.01 | 0.13 | 0.05 | 0.00 | 0.01 | 0.10 | 0.33 |
|  | 17 | NDVI2 | 0.00 | 0.00 | 0.00 | 0.01 | 0.00 | 0.00 | 0.00 | 0.00 | 0.00 | 0.05 | 0.01 | 0.00 | 0.01 | 0.00 | 0.00 | 0.00 | 0.00 | 0.04 | 0.00 | 0.00 | 0.00 | 0.04 | 0.01 | 0.00 | 0.00 | 0.00 | 0.00 | 0.00 | 0.00 | 0.04 | 0.00 | 0.09 | 0.01 | 0.03 | 0.00 | 0.00 | 0.02 | 0.00 | 0.00 | 0.00 | 0.09 | 0.02 | 0.02 | 0.02 | 0.06 |
|  | 15 | REIP | 0.30 | 0.50 | 0.44 | 0.30 | 0.12 | 0.30 | 0.30 | 0.44 | 0.12 | 0.20 | 0.01 | 0.18 | 0.04 | 0.03 | 0.03 | 0.05 | 0.13 | 0.00 | 0.01 | 0.13 | 0.36 | 0.26 | 0.34 | 0.02 | 0.07 | 0.39 | 0.40 | 0.02 | 0.06 | 0.00 | 0.16 | 0.01 | 0.13 | 0.11 | 0.03 | 0.04 | 0.07 | 0.02 | 0.13 | 0.39 | 0.25 | 0.37 | 0.13 | 0.01 | 0.04 |
|  | 16 | REIP | 0.00 | 0.26 | 0.11 | 0.06 | 0.05 | 0.01 | 0.24 | 0.37 | 0.04 | 0.01 | 0.17 | 0.23 | 0.11 | 0.19 | 0.02 | 0.00 | 0.02 | 0.02 | 0.00 | 0.00 | 0.05 | 0.01 | 0.02 | 0.00 | 0.21 | 0.16 | 0.16 | 0.05 | 0.03 | 0.13 | 0.00 | 0.02 | 0.13 | 0.01 | 0.00 | 0.02 | 0.04 | 0.01 | 0.03 | 0.04 | 0.04 | 0.01 | 0.01 | 0.04 | 0.38 |
|  | 17 | REIP | 0.25 | 0.28 | 0.23 | 0.19 | 0.00 | 0.30 | 0.25 | 0.27 | 0.01 | 0.00 | 0.00 | 0.04 | 0.00 | 0.02 | 0.12 | 0.08 | 0.00 | 0.00 | 0.03 | 0.01 | 0.24 | 0.17 | 0.25 | 0.18 | 0.23 | 0.36 | 0.38 | 0.09 | 0.24 | 0.00 | 0.02 | 0.01 | 0.00 | 0.05 | 0.03 | 0.09 | 0.00 | 0.12 | 0.00 | 0.18 | 0.05 | 0.16 | 0.09 | 0.01 | 0.31 |
|  | 15 | WMMRS | 0.33 | 0.41 | 0.51 | 0.39 | 0.11 | 0.18 | 0.37 | 0.45 | 0.12 | 0.16 | 0.00 | 0.18 | 0.06 | 0.01 | 0.03 | 0.21 | 0.25 | 0.00 | 0.01 | 0.13 | 0.32 | 0.19 | 0.37 | 0.14 | 0.08 | 0.31 | 0.43 | 0.05 | 0.08 | 0.01 | 0.09 | 0.00 | 0.26 | 0.14 | 0.05 | 0.00 | 0.22 | 0.03 | 0.14 | 0.42 | 0.26 | 0.42 | 0.23 | 0.01 | 0.04 |
|  | 16 | WMMRS | 0.01 | 0.36 | 0.26 | 0.26 | 0.06 | 0.00 | 0.48 | 0.39 | 0.13 | 0.04 | 0.21 | 0.28 | 0.13 | 0.15 | 0.02 | 0.00 | 0.05 | 0.03 | 0.00 | 0.02 | 0.29 | 0.06 | 0.01 | 0.03 | 0.23 | 0.23 | 0.19 | 0.07 | 0.03 | 0.05 | 0.00 | 0.08 | 0.42 | 0.00 | 0.01 | 0.01 | 0.09 | 0.01 | 0.01 | 0.26 | 0.02 | 0.00 | 0.01 | 0.12 | 0.38 |
|  | 17 | WMMRS | 0.22 | 0.29 | 0.27 | 0.11 | 0.18 | 0.22 | 0.24 | 0.23 | 0.10 | 0.04 | 0.00 | 0.06 | 0.00 | 0.00 | 0.11 | 0.09 | 0.08 | 0.00 | 0.04 | 0.06 | 0.03 | 0.02 | 0.19 | 0.12 | 0.21 | 0.34 | 0.34 | 0.13 | 0.23 | 0.03 | 0.06 | 0.01 | 0.03 | 0.12 | 0.01 | 0.06 | 0.01 | 0.12 | 0.01 | 0.07 | 0.06 | 0.09 | 0.10 | 0.07 | 0.36 |
|  |  |  |  |  |  |  |  |  |  |  |  |  |  |  |  |  |  |  |  |  |  |  |  |  |  |  |  |  |  |  |  |  |  |  |  |  |  |  |  |  |  |  |  |  |  |  |  |
| RMSE (calibration) | 15 | PLSR | 746 | 720 | 365 | 112 | 188 | 503 | 71 | 274 | 222 | 37 | 0.074 | 618 | 495 | 0.036 | 0.019 | 1.56 | 2.46 | 6.9 | 3.5 | 3.0 | 3.7 | 4.1 | 9.6 | 1.1 | 1.2 | 7.9 | 9.0 | 1.5 | 2.5 | 0.07 | 0.15 | 0.06 | 0.08 | 0.03 | 0.08 | 0.08 | 0.04 | 0.01 | 2.3 | 3.9 | 3.5 | 7.9 | 0.018 | 8.0 | 1.8 |
|  | 16 | PLSR | 864 | 688 | 397 | 174 | 149 | 628 | 74 | 360 | 149 | 39 | 0.091 | 754 | 741 | 0.042 | 0.014 | 1.77 | 2.87 | 4.1 | 2.5 | 2.9 | 6.0 | 7.2 | 13.4 | 1.3 | 1.4 | 8.9 | 9.1 | 1.5 | 3.2 | 0.07 | 0.18 | 0.08 | 0.09 | 0.01 | 0.08 | 0.06 | 0.07 | 0.01 | 1.7 | 6.1 | 7.4 | 12.8 | 0.017 | 14.3 | 1.7 |
|  | 17 | PLSR | 601 | 719 | 312 | 221 | 168 | 426 | 135 | 358 | 157 | 57 | 0.062 | 753 | 569 | 0.041 | 0.012 | 1.27 | 2.05 | 5.0 | 3.1 | 4.2 | 7.5 | 6.4 | 12.6 | 1.8 | 2.7 | 7.5 | 10.4 | 1.4 | 4.9 | 0.10 | 0.17 | 0.07 | 0.07 | 0.04 | 0.07 | 0.05 | 0.04 | 0.01 | 3.5 | 7.1 | 5.4 | 10.3 | 0.016 | 10.2 | 2.0 |
|  | 15 | NDVI2 | 791 | 810 | 445 | 164 | 196 | 524 | 100 | 312 | 228 | 40 | 0.074 | 632 | 499 | 0.043 | 0.022 | 1.89 | 3.32 | 6.9 | 4.0 | 3.2 | 5.1 | 4.2 | 10.5 | 1.4 | 1.4 | 9.6 | 10.3 | 1.5 | 3.2 | 0.07 | 0.15 | 0.06 | 0.12 | 0.03 | 0.10 | 0.08 | 0.05 | 0.02 | 2.7 | 4.4 | 3.7 | 8.8 | 0.024 | 10.1 | 1.8 |
|  | 16 | NDVI2 | 863 | 827 | 467 | 227 | 162 | 631 | 124 | 396 | 157 | 46 | 0.094 | 813 | 745 | 0.044 | 0.015 | 1.93 | 2.86 | 5.0 | 2.9 | 2.9 | 8.5 | 7.5 | 13.4 | 2.4 | 1.9 | 9.5 | 10.8 | 1.5 | 4.2 | 0.08 | 0.19 | 0.09 | 0.17 | 0.02 | 0.08 | 0.06 | 0.07 | 0.02 | 2.5 | 8.7 | 7.9 | 12.8 | 0.022 | 15.0 | 2.0 |
|  | 17 | NDVI2 | 704 | 907 | 354 | 220 | 218 | 439 | 164 | 433 | 187 | 59 | 0.062 | 757 | 570 | 0.041 | 0.015 | 1.31 | 2.35 | 5.1 | 3.1 | 4.7 | 7.6 | 6.4 | 12.8 | 1.8 | 2.9 | 7.8 | 11.1 | 1.5 | 5.1 | 0.10 | 0.17 | 0.07 | 0.07 | 0.04 | 0.08 | 0.05 | 0.04 | 0.01 | 3.9 | 7.2 | 5.4 | 10.4 | 0.017 | 10.5 | 2.2 |
|  | 15 | REIP | 844 | 911 | 468 | 159 | 195 | 576 | 98 | 367 | 232 | 41 | 0.075 | 641 | 500 | 0.043 | 0.022 | 1.83 | 3.31 | 6.9 | 4.0 | 3.2 | 5.1 | 4.5 | 10.9 | 1.4 | 1.5 | 10.6 | 11.5 | 1.5 | 3.3 | 0.07 | 0.16 | 0.06 | 0.11 | 0.03 | 0.10 | 0.08 | 0.05 | 0.02 | 2.7 | 4.5 | 3.8 | 9.1 | 0.024 | 10.2 | 1.8 |
|  | 16 | REIP | 862 | 798 | 432 | 217 | 158 | 623 | 117 | 417 | 157 | 45 | 0.094 | 801 | 756 | 0.045 | 0.015 | 1.92 | 2.87 | 5.0 | 2.9 | 2.9 | 8.1 | 7.5 | 13.5 | 2.4 | 1.8 | 9.1 | 10.4 | 1.5 | 4.2 | 0.08 | 0.19 | 0.09 | 0.17 | 0.02 | 0.08 | 0.06 | 0.07 | 0.02 | 2.5 | 8.3 | 7.8 | 12.8 | 0.022 | 14.6 | 2.1 |
|  | 17 | REIP | 812 | 1070 | 404 | 244 | 217 | 526 | 189 | 505 | 188 | 58 | 0.062 | 773 | 569 | 0.041 | 0.016 | 1.37 | 2.35 | 5.0 | 3.1 | 4.7 | 8.7 | 6.8 | 14.8 | 1.9 | 3.3 | 9.7 | 14.1 | 1.6 | 5.8 | 0.10 | 0.17 | 0.07 | 0.07 | 0.04 | 0.08 | 0.05 | 0.04 | 0.01 | 3.9 | 8.0 | 5.3 | 11.3 | 0.018 | 10.5 | 2.5 |
|  | 15 | WMMRS | 778 | 900 | 344 | 230 | 197 | 464 | 165 | 443 | 178 | 58 | 0.062 | 750 | 570 | 0.043 | 0.015 | 1.31 | 2.26 | 5.1 | 3.1 | 4.6 | 8.6 | 6.9 | 10.2 | 1.8 | 2.9 | 7.9 | 11.4 | 1.5 | 5.1 | 0.10 | 0.17 | 0.07 | 0.07 | 0.04 | 0.08 | 0.05 | 0.04 | 0.01 | 3.9 | 7.7 | 5.4 | 10.9 | 0.017 | 10.2 | 2.1 |
|  | 16 | WMMRS | 862 | 769 | 424 | 201 | 161 | 632 | 103 | 392 | 149 | 45 | 0.092 | 789 | 735 | 0.044 | 0.015 | 1.93 | 2.81 | 5.0 | 2.9 | 2.9 | 7.3 | 7.3 | 13.5 | 2.4 | 1.9 | 9.1 | 10.7 | 1.5 | 4.2 | 0.08 | 0.19 | 0.09 | 0.14 | 0.02 | 0.08 | 0.06 | 0.07 | 0.02 | 2.5 | 7.6 | 8.0 | 12.8 | 0.022 | 14.4 | 2.0 |
|  | 17 | WMMRS | 718 | 900 | 344 | 230 | 197 | 464 | 165 | 443 | 178 | 58 | 0.062 | 750 | 570 | 0.041 | 0.015 | 1.31 | 2.26 | 5.1 | 3.1 | 4.6 | 8.6 | 6.9 | 13.4 | 1.8 | 2.9 | 7.9 | 11.4 | 1.5 | 5.1 | 0.10 | 0.17 | 0.07 | 0.07 | 0.04 | 0.08 | 0.05 | 0.04 | 0.01 | 3.9 | 7.7 | 5.4 | 10.9 | 0.017 | 10.2 | 2.1 |


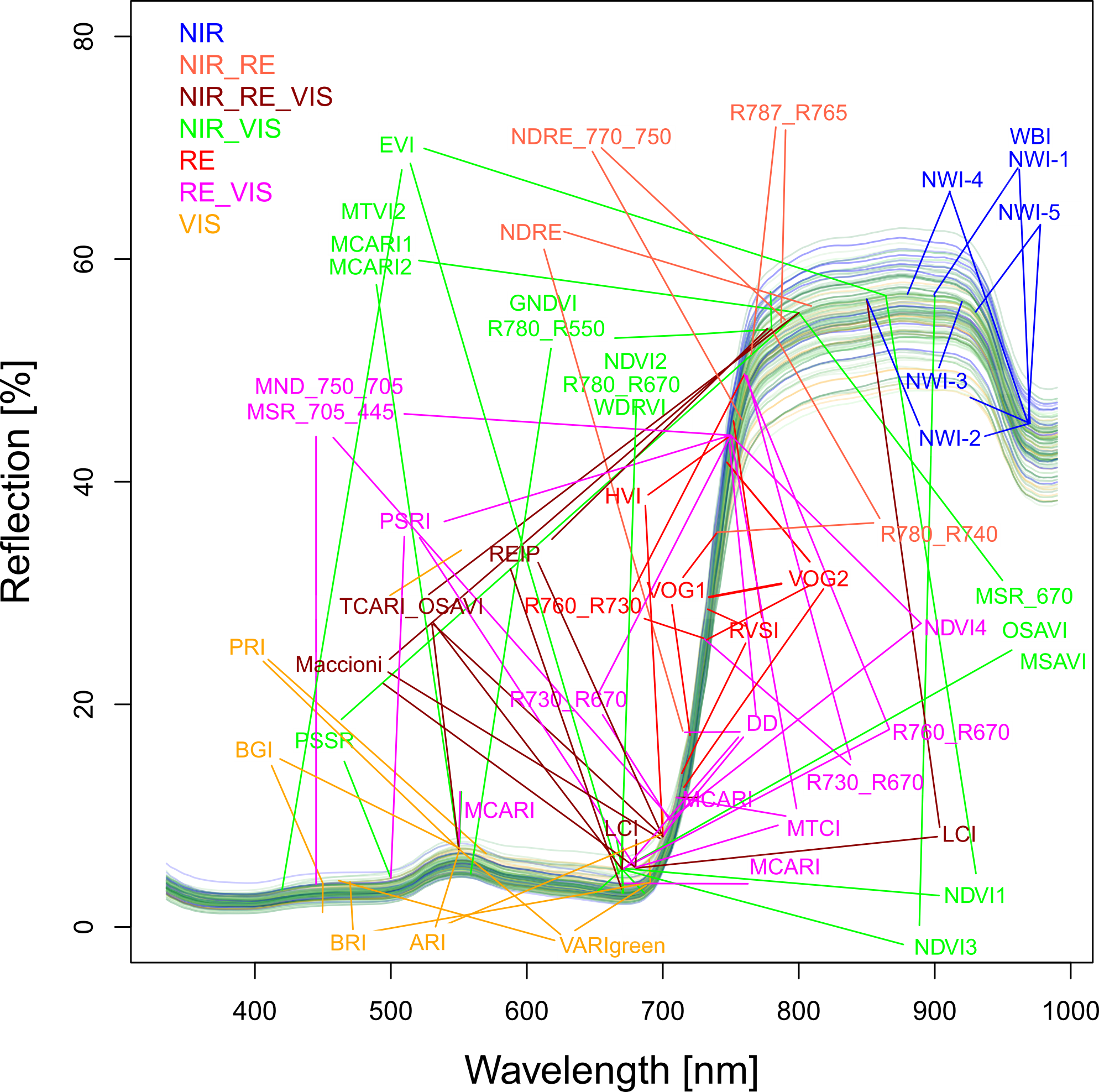


Supplementary Figure 1: Spectra acquired on 21 June 2017 with band position of indices, colored by spectral regions: Near infrared (NIR: > 765 nm), visible (VIS: < 700 nm), extended red edge (RE: 700–765 nm). The spectra are colored by grain yield from yellow (lowest yield) to green (medium yield) and blue (highest yield).


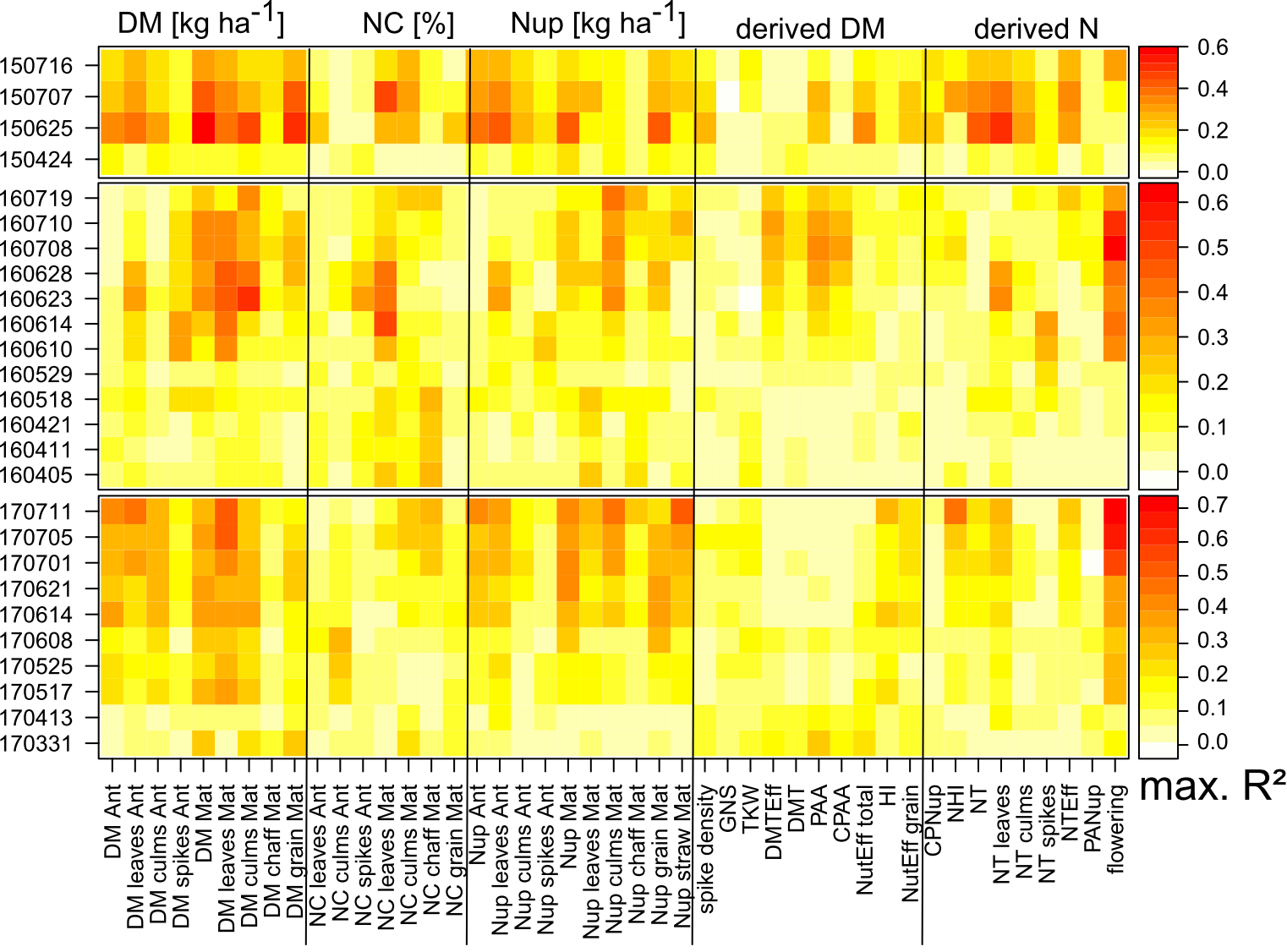


Supplementary Figure 2: Maximum coefficients of determination (R²) across indices by traits and measurement dates in the three years. See Table 1 for trait abbreviations. Dates are indicated as year-month-day.


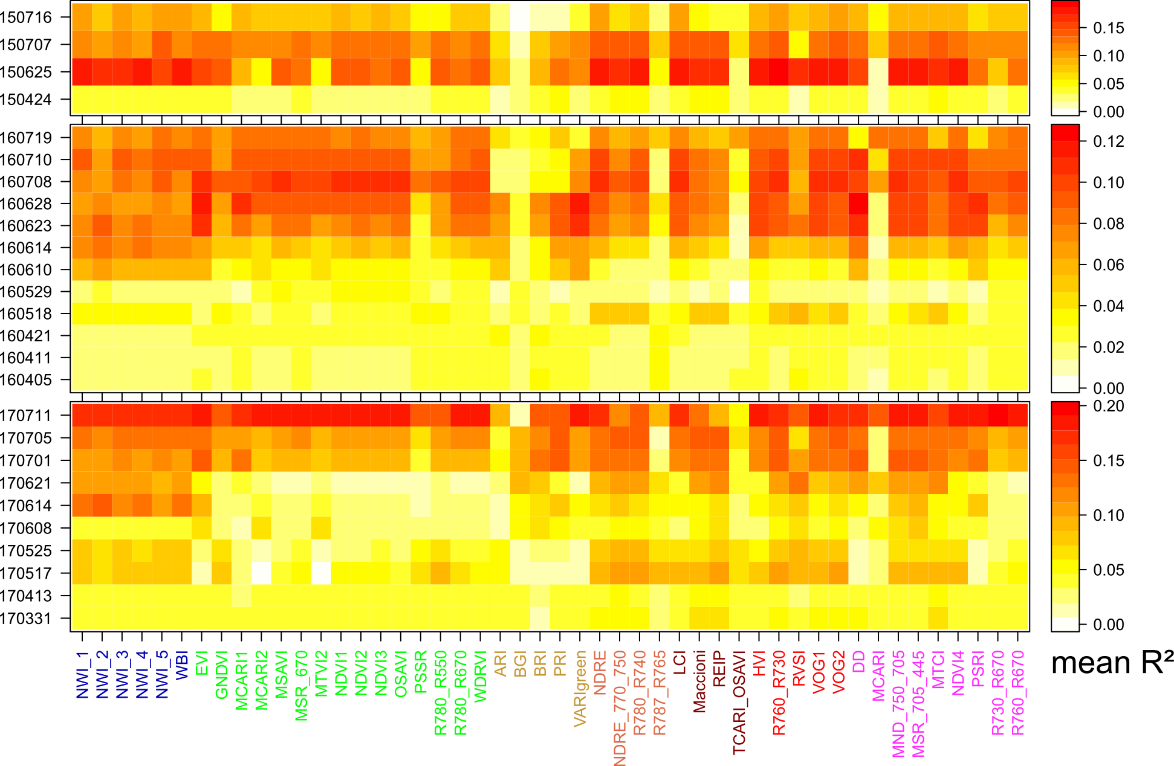


Supplementary Figure 3: Mean coefficients of determination (R²) across traits by SVIs and measurement dates in the three years. Indices are colored according to the included spectral regions (Supplementary Figure 1): NIR (blue), NIR/VIS (green), VIS (orange), NIR/RE (light red), NIR/RE/VIS (brown), RE (red) and RE/VIS (purple). See Table 2 for index abbreviations and index grouping. Dates are indicated as year/month/day.


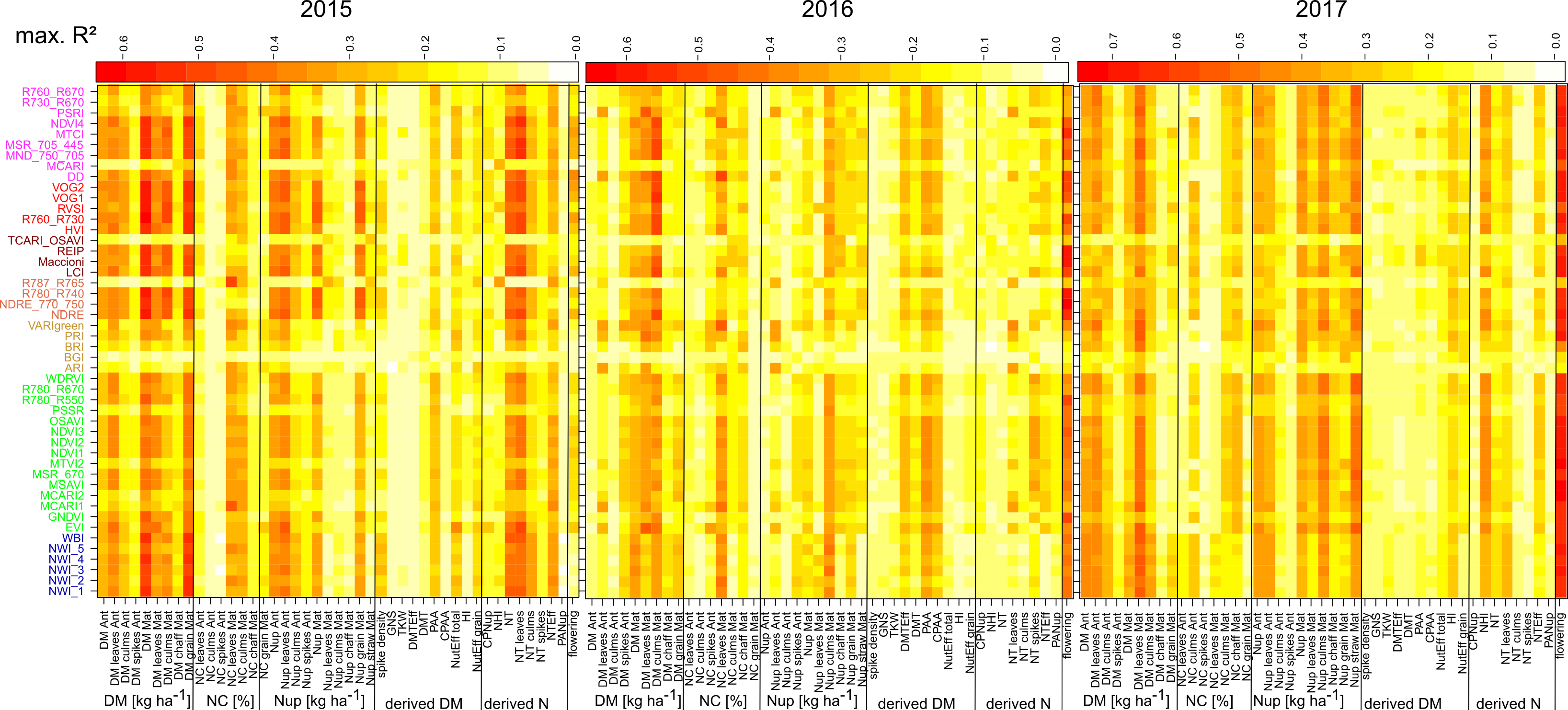


Supplementary Figure 4: Maximum coefficients of determination (R²) calculated across the R²-values of the different measurement dates (n = 4 in 2015, n = 12 in 2016 and n = 10 in 2017) by trait*SVI combinations. Indices are colored according to the included spectral regions: NIR (blue), NIR/VIS (green), VIS (orange), NIR/RE (light red), NIR/RE/VIS (brown), RE (red), RE/VIS (purple).


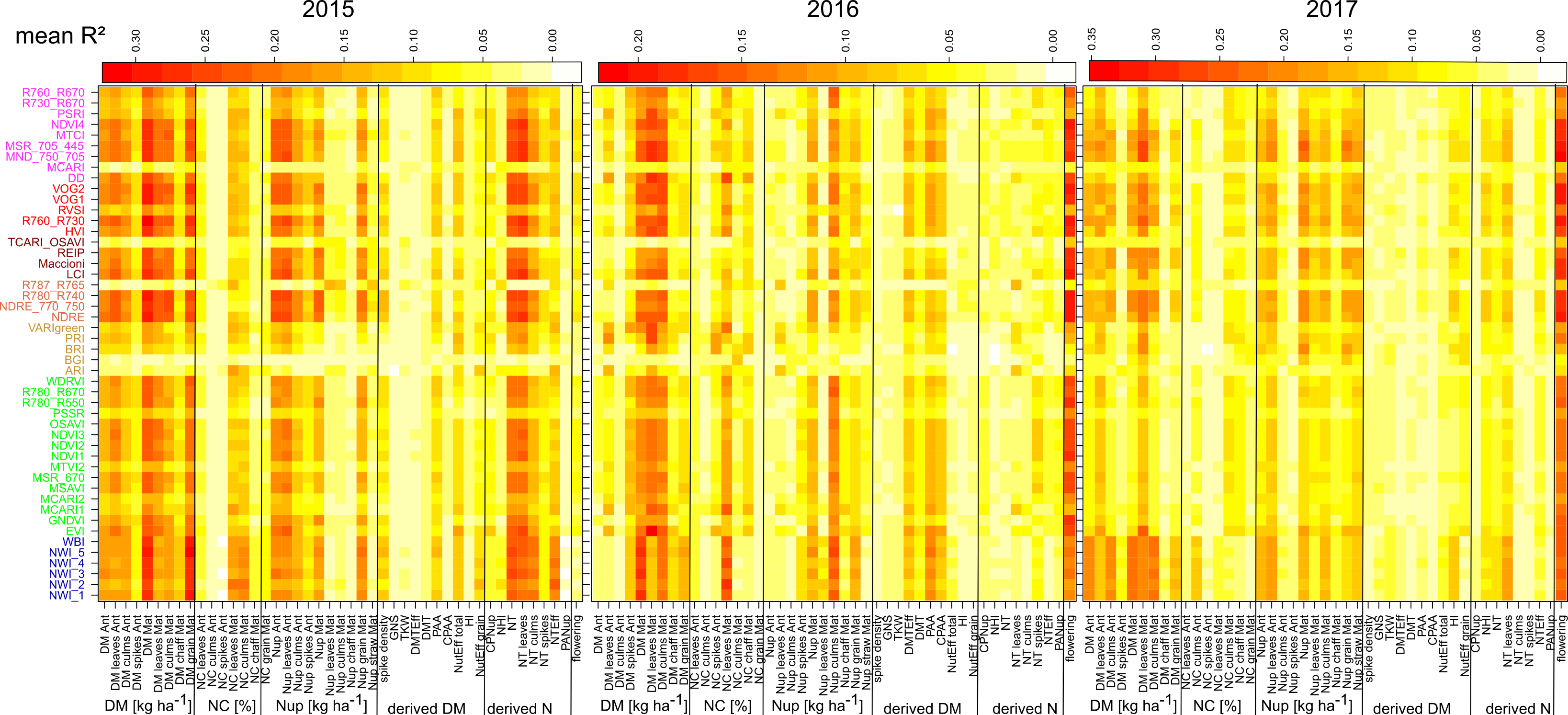


Supplementary Figure 5: Mean coefficients of determination (R²) from R² values of the different measurement dates (n = 4 in 2015, n = 12 in 2016 and n = 10 in 2017) by trait*SVI combinations. Indices are colored according to the included spectral regions (Supplementary Figure 1): NIR (blue), NIR/VIS (green), VIS (orange), NIR/RE (light red), NIR/RE/VIS (brown), RE (red) and RE/VIS (purple).


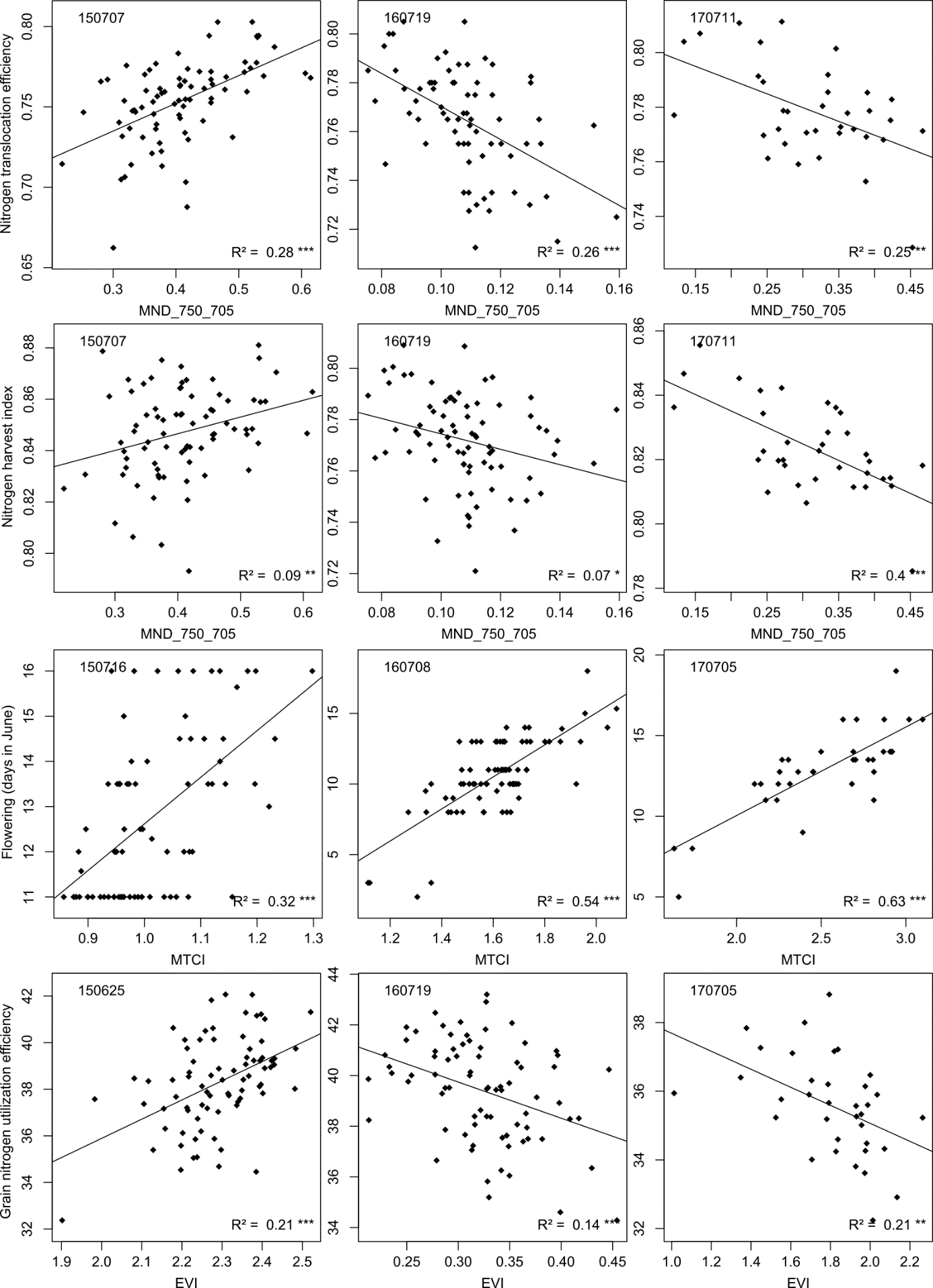
Supplementary Figure 6: Relationships of the rank-based best indices on the trait-specific most suitable dates with selected traits: Nitrogen translocation efficiency, nitrogen harvest index, flowering date (days in June) and grain nitrogen utilization efficiency. For nitrogen harvest index, the same index and dates are shown as for nitrogen translocation efficiency instead.

Supplementary Figure 7: Comparison of coefficients of determination (R²) of water band indices for grain yield during the grain filling phase.


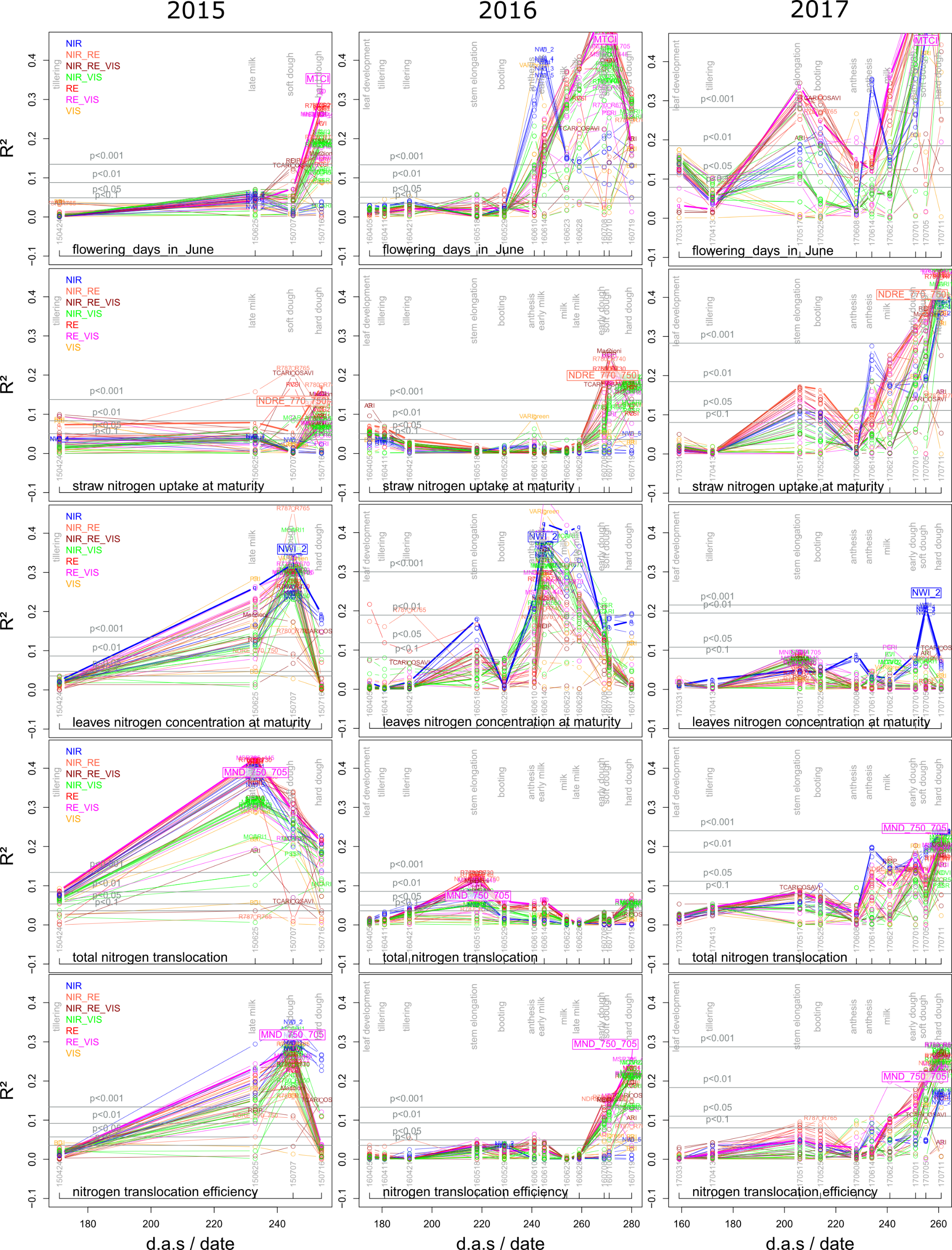


Supplementary Figure 8: Seasonal R²-values for selected traits. See caption of Figure 3 for details.


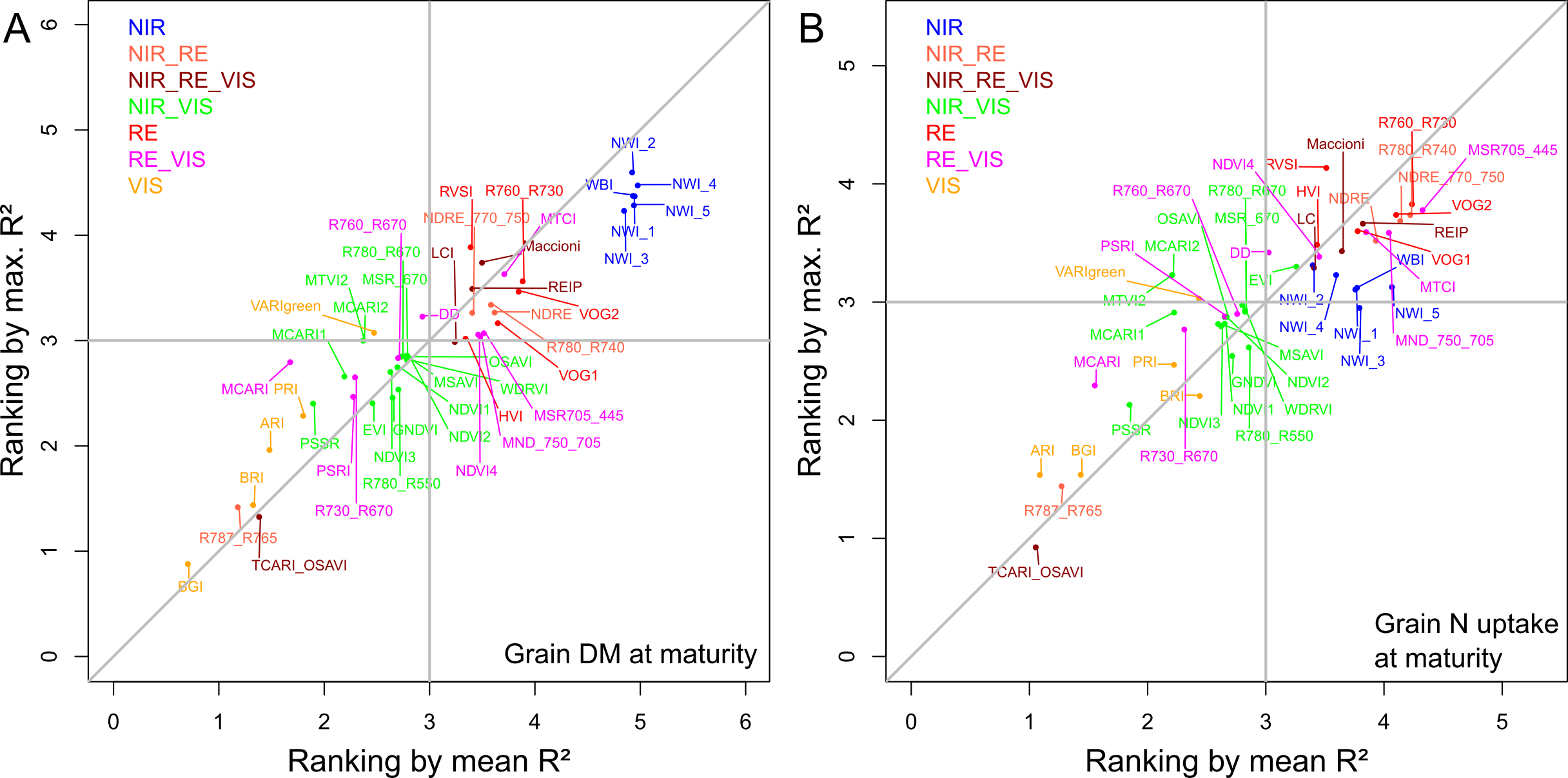


Supplementary Figure 9: Across-years mean-based (x-axis) and maximum-based (y-axis) quantitative index rankings for grain yield (left) and grain N uptake (right). Indices are colored according to the included spectral regions. Gray lines mark average rankings (3).


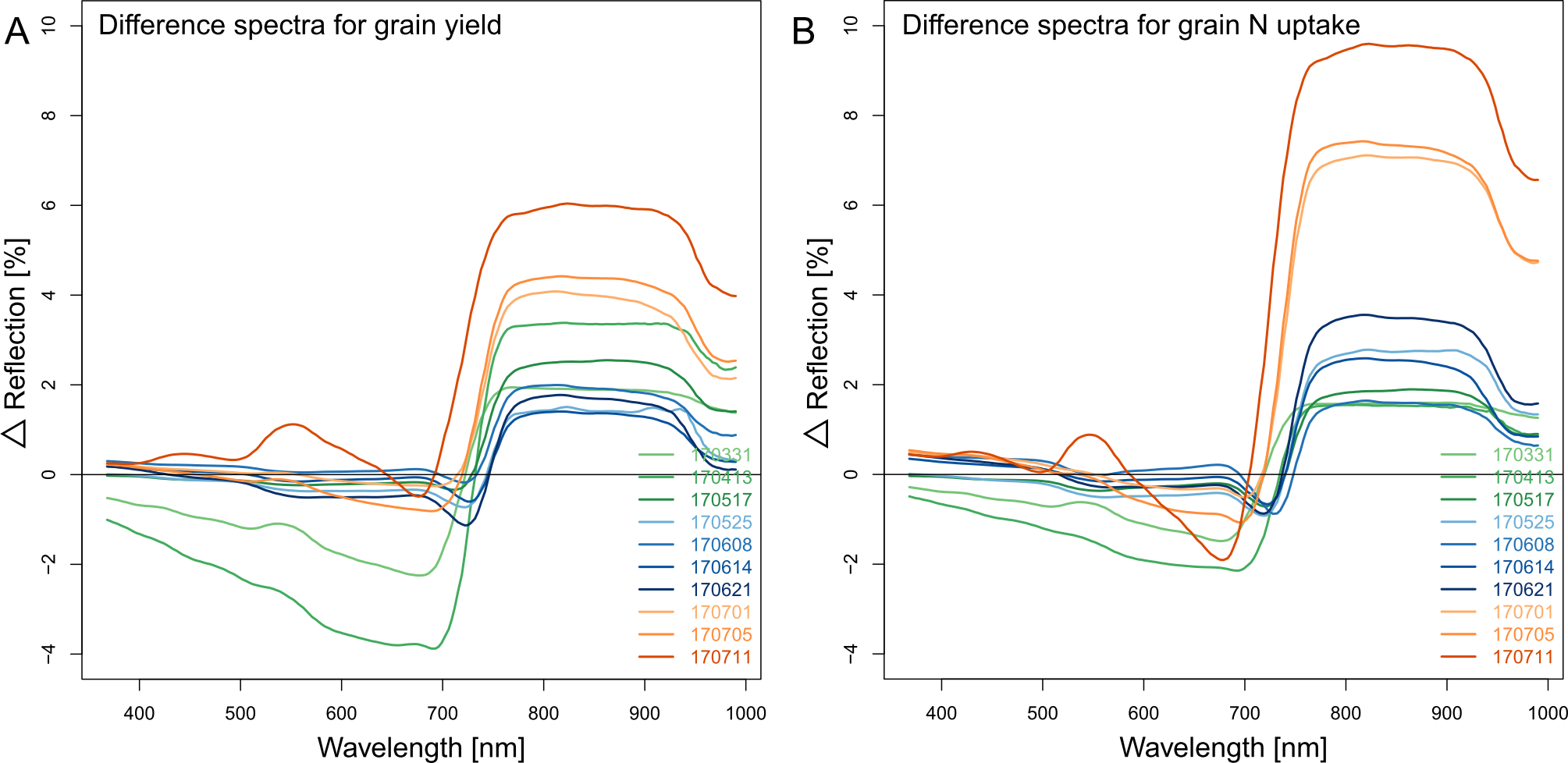


Supplementary Figure 10**:** Spectra by measurement dates in 2017: (A) difference spectra between the reflection of the 1/9 of highest yielding and 1/9 of lowest yielding plots; (B) difference spectra between the spectra of the 1/9 highest Nup-yielding and 1/9 of lowest Nup-yielding plots.


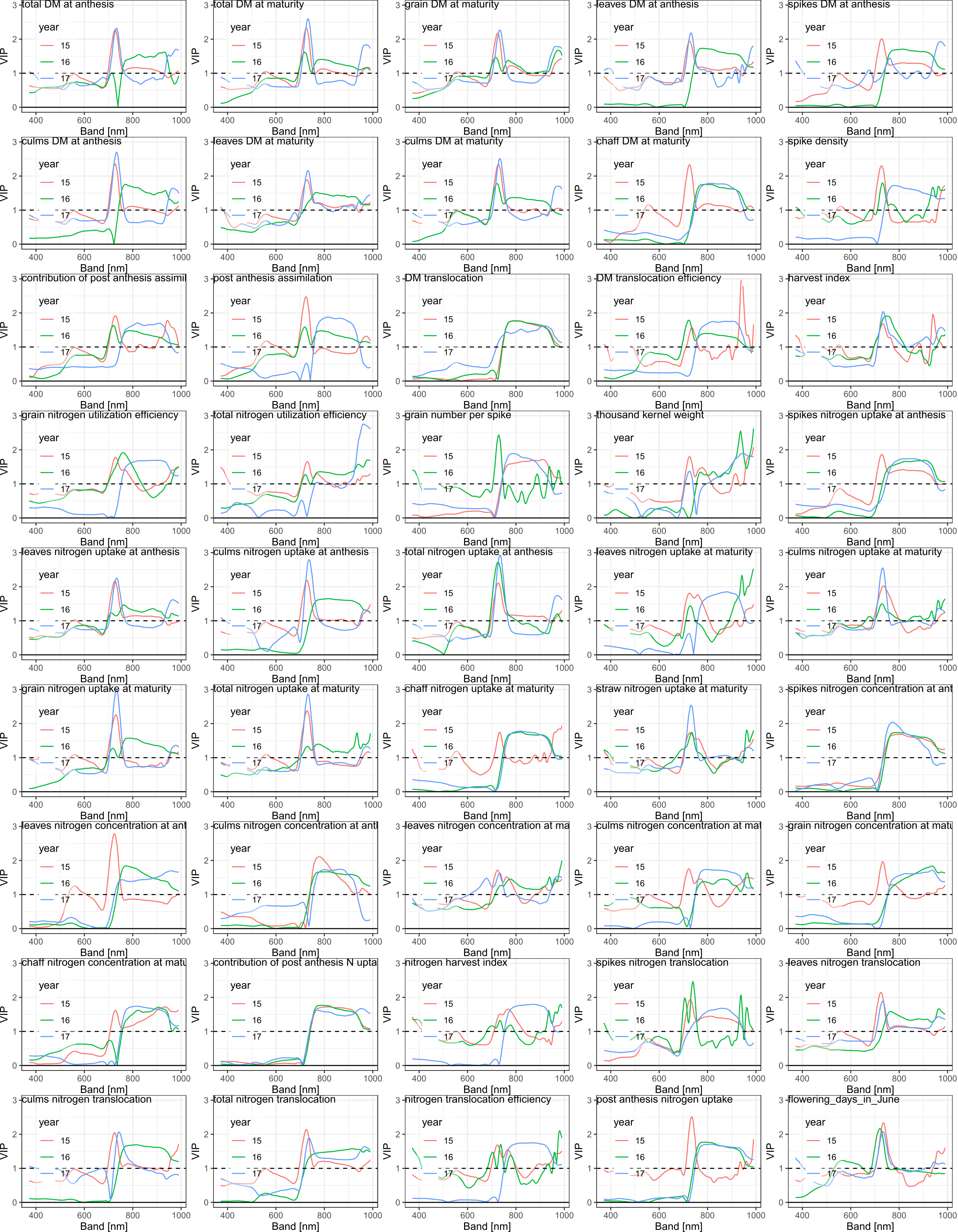


Supplementary Figure 11: Variable Importance in Projection (VIP) of PLSR models by traits in the three years 2015 (15), 2016 (16) and 2017 (17). VIP-values > 1 (dashed lines) are considered as influential bands. Spectra were smoothed prior to the analysis.
